# Chemical‐Biological Fixation for Enhanced Bone Augmentation in Guided Bone Regeneration

**DOI:** 10.1002/advs.76877

**Published:** 2026-07-30

**Authors:** Yu‐zhu Wang, Gao‐peng Dang, Zhi‐ting Li, Zhi‐hong Feng, Que Bai, Jia‐xin Hao, Xiao‐qing Cao, Tao Ye, Jing Li, Franklin R. Tay, Malcolm Xing, Ming Fang, Jun‐ting Gu, Li‐na Niu

**Affiliations:** ^1^ State Key Laboratory of Oral & Maxillofacial Reconstruction and Regeneration National Clinical Research Center for Oral Diseases School of Stomatology The Fourth Military Medical University Xi'an Shaanxi China; ^2^ The Third Affiliated Hospital of Henan Medical University Xinxiang Henan China; ^3^ Dental College of Georgia Augusta University Augusta Georgia USA; ^4^ Department of Mechanical Engineering University of Manitoba Winnipeg Manitoba Canada; ^5^ National Translational Science Center for Molecular Medicine Department of Cell Biology State Key Laboratory of Cancer Biology Medical Innovation Center The Fourth Military Medical University Xi'an Shaanxi China

**Keywords:** bone substitute materials, clot mechanics, fibrin blood clot, guided bone regeneration, self‐fixed barrier membrane

## Abstract

Stable fixation of barrier membranes is essential for achieving effective bone augmentation during guided bone regeneration (GBR). Conventional fixation techniques, such as membrane nails and sutures, provide suboptimal results and may lead to the displacement of bone substitute materials, eventually compromising regenerative outcomes. To address this, a thermosensitive injectable adhesive, M2N2ACa, was developed to solidify upon exposure to a 36°C wet environment. This material was found to facilitate robust adhesion between barrier membranes and moist bone surfaces at 36°C, achieving an adhesive strength of 227.86 kPa. In addition to its mechanical bonding capabilities, M2N2ACa can release calcium ions to enhance clot density and mechanical strength, thereby reinforcing the surrounding blood clot and stabilizing the bone substitutes. This dual mechanism of mechanical fixation through membrane bonding and biological reinforcement via clot modulation substantially improves graft retention and promotes bone augmentation. These findings highlight the value of M2N2ACa as a promising barrier membrane adhesive with the potential to simplify clinical procedures and improve GBR outcomes.

## Graphical abstract

M2N2ACa is an innovative injectable thermoresponsive barrier membrane adhesive to achieve reliable bone graft fixation. It provides strong wet adhesion and promotes the bio‐stabilization mediated by coagulation through controlled calcium release. This dual‐fixation strategy combines external chemical fixation and internal biological fixation via clot mechanics, providing an illuminating direction for future guided bone regeneration techniques.

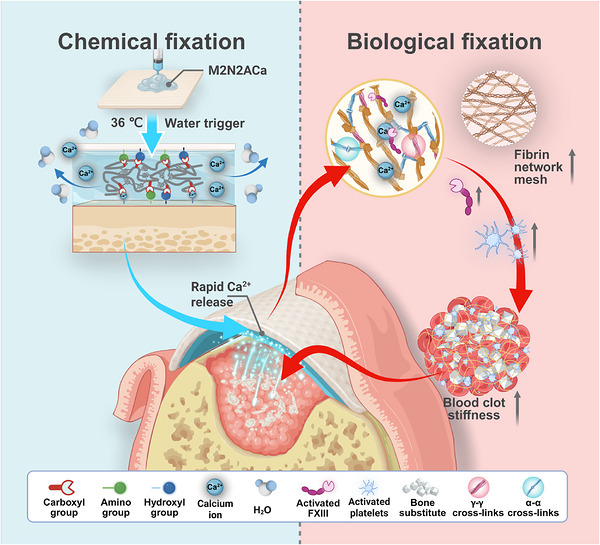

## 1 Introduction

Guided bone regeneration (GBR) is a widely utilized technique for augmenting the alveolar bone in both implant and periodontal therapy. A key component of GBR is the application of a barrier membrane, which serves to isolate the bone graft from surrounding soft tissues and preserve space for new bone formation [1, 2]. Currently, the combination of particulate bone graft and bioabsorbable barrier membranes represents a mainstream clinical approach. Among various options, collagen membranes have become a preferred choice due to their excellent handling properties, easy adaptation to diverse defect contours, and superior biocompatibility [3, 4]. To be effective, the membrane must remain immobile and closely adapted to the defect site to prevent displacement of the graft material during healing [5, 6]. Achieving mechanical stability is particularly challenging in the oral environment, especially when particulate graft materials, such as bone granules or powder, are used. Inadequate membrane fixation can result in graft movement, which interferes with early tissue integration, delays osteogenesis, and increases the risk of infection [7]. Clinical studies have reported bone graft displacement rates as high as 30% in cases of insufficient fixation, leading to compromised bone augmentation outcomes [8].

Current fixation techniques, including titanium nails, resorbable pins, and suture anchorage, present some inherent limitations [9, 10]. These techniques often involve complex intraoperative handling, prolong the surgical duration, and may cause trauma or injury to adjacent anatomical structures [7, 11]. Moreover, tucking the membrane under mucoperiosteal flaps does not reliably immobilize the membrane and is associated with higher rates of displacement [12]. Collectively, these disadvantages highlight the need for alternative fixation strategies that offer simplicity, biocompatibility, and effectiveness under physiological conditions.

**FIGURE 1:**
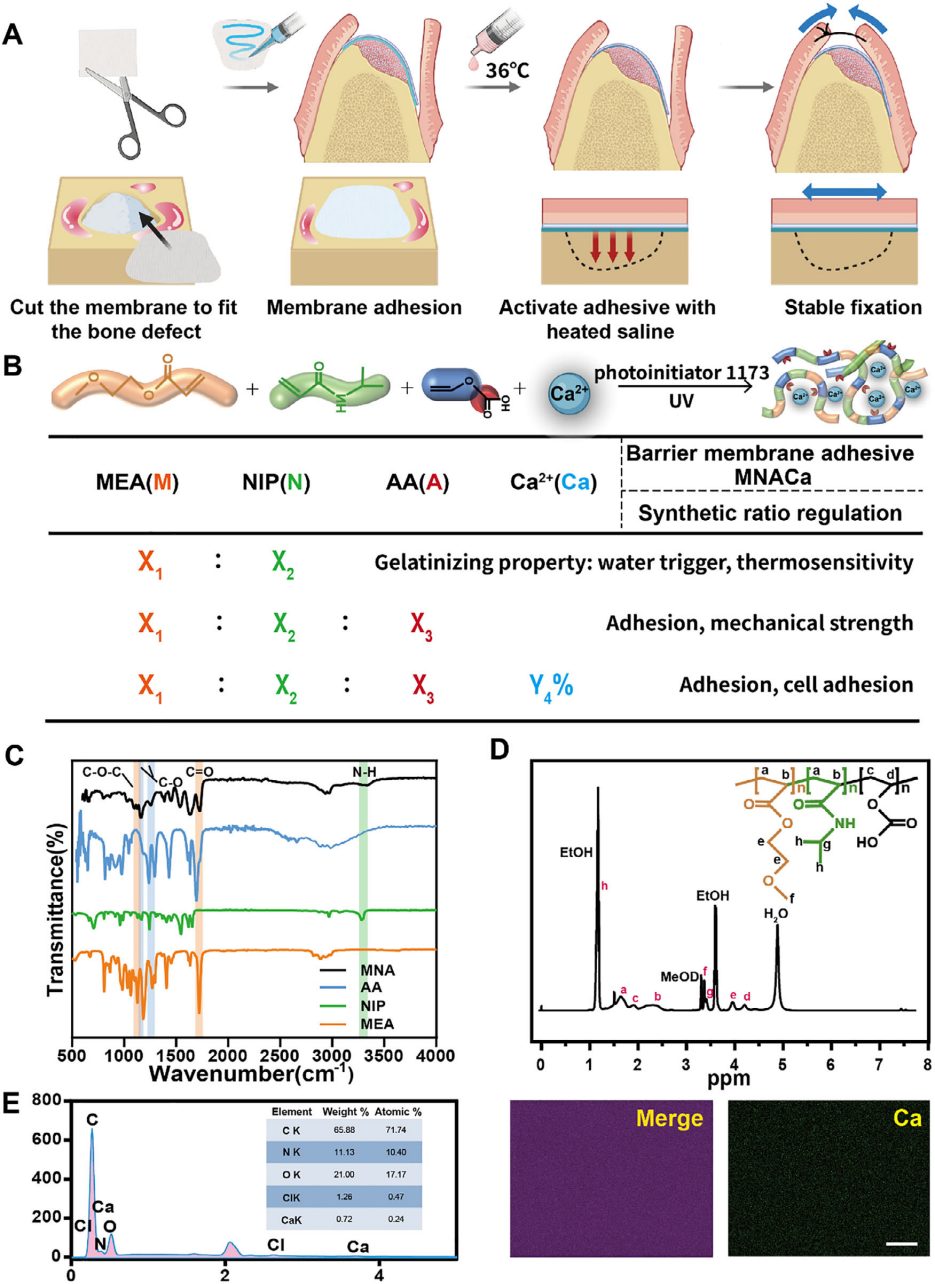
The preparation of MNACa. (A) Schematic of the surgical application process. (B) Molecular design and synthetic route of methacryloxyethyl methacrylate‐N‐isopropylacrylamide—acrylic acid—calcium adhesives (MNACa). (C) Infrared spectra comparing MEA, NIP, AA, and MNA samples. (D) ^1^H NMR spectra of MNA in MeOD. (E) EDX mapping illustrating the elemental composition and spatial distribution of Ca^2+^ across the MNACa surface. Scale bar: 20 µm. Schematic generated with BioRender.

In recent years, tissue adhesives have emerged as a promising class of materials for minimally invasive membrane fixation [13, 14]. Their advantages include ease of application, reduced tissue trauma, and the ability to conform to irregular anatomical surfaces. However, no adhesive system to date has specifically been engineered or clinically approved for securing barrier membranes in GBR procedures [15, 16, 17]. A major challenge in designing such adhesives lies in achieving strong and durable bonding in wet environments such as the oral cavity, where saliva, blood, and tissue fluids interfere with interfacial adhesion (Figure 1A) [18, 19, 20]. Additionally, such adhesives also need to exhibit controlled curing behavior and be biocompatible with biological tissues [21].

In addition to external membrane fixation, the structural and mechanical properties of the blood clot formed at the defect site also play an important role in graft stabilization. The blood clot functions as a provisional matrix, binding to bone particles and initiating tissue regeneration [7, 22]. The ability of the clot to withstand dislodging forces and maintain the spatial integrity of the graft is governed by its viscoelastic properties, which in turn influence the clinical success of bone augmentation [23]. Despite its importance, the role of the blood clot in graft fixation has received limited attention in the realm of GBR material design.

To address these challenges, an injectable adhesive, M2N2ACa, was developed. This adhesive integrated thermoresponsive solidification with wet‐surface adhesion and calcium ion release to support clot formation and stabilization. It was formulated using methoxyethyl acrylate (MEA), N‐isopropylacrylamide (NIP), and acrylic acid (AA), with calcium ions serving both as a crosslinking agent for the adhesive and a bioactive component in the blood clots. This adhesive was designed to provide dual‐function fixation via the mechanical anchoring of the barrier membrane to wet bone surfaces and the biological reinforcement of the underlying blood clot to minimize graft displacement.

This study evaluated whether the combined effects of external fixation via membrane adhesion and internal reinforcement via clot stabilization could improve graft retention and enhance bone regeneration in GBR. The central hypothesis was that an injectable, thermosensitive adhesive capable of strong wet adhesion and calcium‐mediated clot reinforcement would provide superior stabilization of bone substitutes compared to conventional fixation methods. To test this hypothesis, the physicochemical properties, biocompatibility, and fixation performance of M2N2ACa were systematically assessed under dynamic conditions in vivo.

## 2 Results and Discussion

### 2.1 Preparation of MNACa via Free Radical Polymerization

Three monomers—MEA, NIP, and AA—were polymerized via photoinitiated free radical polymerization to produce the polymer backbone of the MNACa adhesive hydrogel [24]. Calcium ions (Ca^2+^) were introduced for cross‐linking during the final stage of the reaction to enhance the internal connectivity of the polymer network [25]. Adhesion to wet bone surfaces was enhanced due to the combined properties of the three monomers: MEA conferred hydrophobicity, NIP enabled thermosensitivity, and AA provided functional groups that facilitate interfacial bonding under wet conditions [26, 27, 28, 29].

The systematic adjustment of the molar ratios of these components led to the development of different formulations (Figure 1B). For example, the M2N2ACa formulation contained MEA, NIP, and AA in a molar ratio of 1:2:2, with Ca^2+^ introduced at a 1% mass fraction. During the initial polymerization phase, MEA, NIP, and AA were polymerized to produce copolymer chains (MNA). Copolymerization was confirmed using Fourier‐transform infrared spectroscopy (FTIR) by identifying characteristic absorption peaks corresponding to each monomer in the resulting MNA polymer (Figure 1C). The N─H stretching vibration of NIP appeared at 3335 cm^−1^, the C═O stretching vibration of MEA at 1728 cm^−1^, and the C─O─C vibration at 1128 cm^−1^. Following polymerization, a shift in the C─O stretching vibration of AA was observed at 1163 cm^−1^.

Copolymer formation was further verified through ^1^H nuclear magnetic resonance (NMR) spectroscopy (Figure 1D). Successful polymerization was confirmed based on the disappearance of hydrogen signals in the low‐field region (δH 4.8–6.8) that corresponded to the presence of vinyl double bonds, as well as the emergence of multiple new signals in the high‐field range (δH 1.6–2.4). The molecular weight of the synthesized MNA copolymer was determined by gel permeation chromatography (GPC). The copolymer exhibited a weight‐average molecular weight of around 80 000 g mol^−1^ and the polydispersity index (PDI) of about 4.34 (Figure S1), indicating the formation of a high molecular weight polymeric network. Surface elemental mapping through energy‐dispersive x‐ray spectroscopy (EDS) demonstrated the uniform distribution of Ca^2+^ throughout the hydrogel network (Figure 1E).

Moreover, MNACa hydrogels exhibited good injectability before gelation and rapid temperature‐sensitive gelation in aqueous environments (Figure S2 and Video S1), forming mechanically robust hydrogels with strong adhesive properties (Figure S3). The adhesive hydrogels also remained stable in wet environments after gelation (Figure S4). These findings confirmed the suitability of MNACa for barrier membrane adhesion in GBR applications [30]. Thus, subsequent experiments focused on optimizing the formulation ratios of MNACa to improve performance.

### 2.2 Impact of the Hydrophobic/Thermosensitive Moiety Ratio (M:N) on Gelation Performance

**FIGURE 2:**
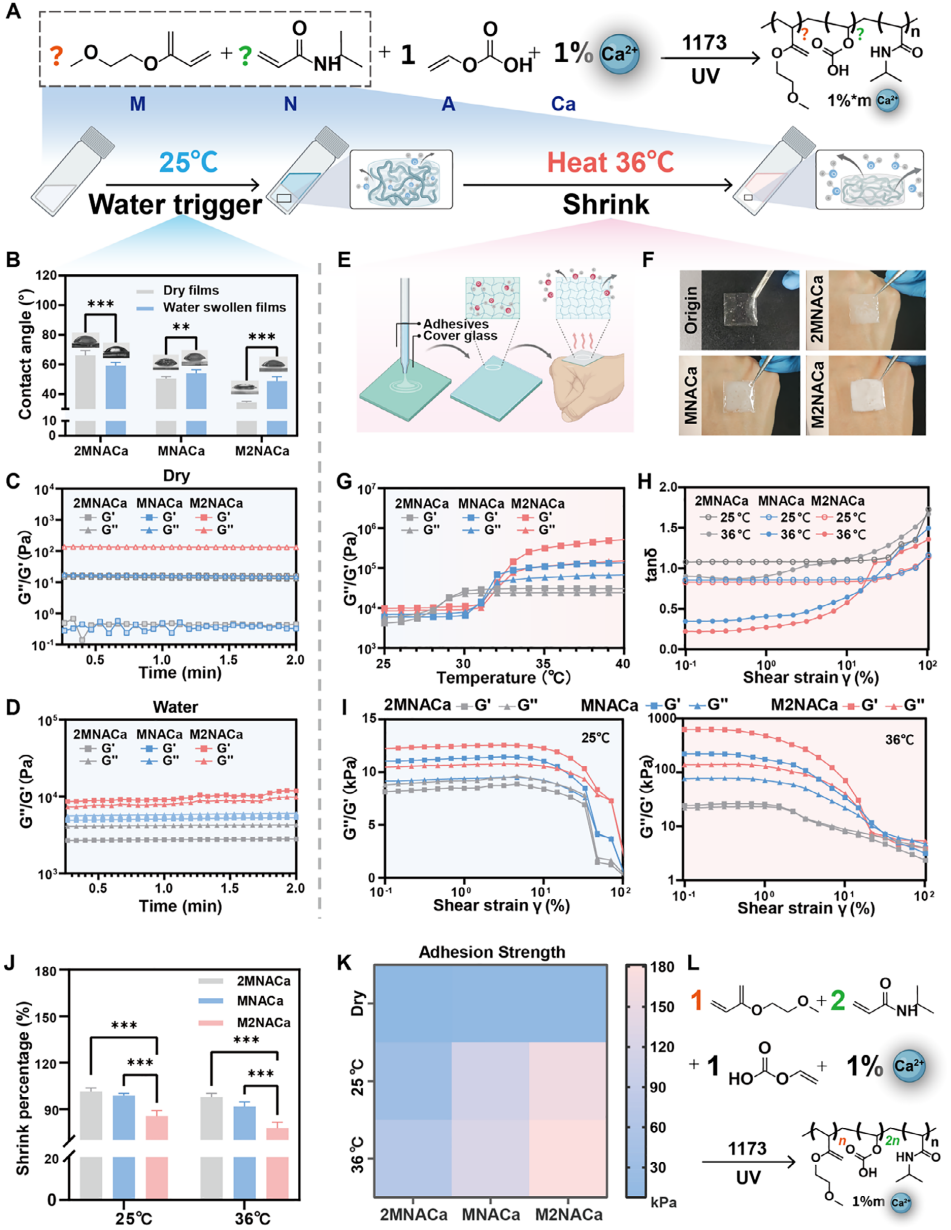
Comparison and analysis of underwater gelation properties of 2MNACa, MNACa, and M2NACa with different M: N synthesis ratios. (A) Synthetic route of 2MNACa, MNACa, and M2NACa. m represents the sum of the total monomer mass. (B) Representative images and water contact angles of dry and water‐swollen films of 2MNACa, MNACa, and M2NACa at 25°C (*n* = 4). The viscoelastic behavior of 2MNACa, MNACa, and M2NACa before (C) and after (D) contact with water. (E) Schematics and (F) representative photograph of the temperature‐triggered phase transitions. (G) Temperature‐dependent modulus curves of 2MNACa, MNACa, and M2NACa. (H, I) The viscoelastic behavior of 2MNACa, MNACa, and M2NACa from 25°C to 36°C. (J) Underwater shrink percentage ratio of 2MNACa, MNACa, and M2NACa (*n* = 3). (K) Shear strength of three adhesives in the dry, 25°C wet, and 36°C wet (*n* = 6). (L) Screening results of the M: N Synthesis Ratio. Data shown as mean ± standard deviation (*n* ≥ 3; ^**^ *p* < 0.005, ^***^ *p* < 0.001). The different background colors in the figure represent different experimental temperatures (Light blue represents 25°C, light red represents 36°C). Schematic generated with BioRender.

Gelation behavior is critical for achieving robust cohesion in hydrogels, which directly contributes to strong adhesive strength in wet environments [24]. Accordingly, adhesive hydrogels containing different ratios of hydrophobic and thermosensitive moieties (MEA and NIP, respectively) were prepared and evaluated. Specifically, the MEA:NIP ratio was systematically adjusted (i.e., 2MNACa, MNACa, and M2NACa) to investigate how gelation is influenced by compositional variation (Figure 2A).

Water contact angle measurements in both the dry and swollen states (25°C) demonstrated that the M:N ratio could modulate the interfacial hydration behavior of the hydrogel (Figure 2B) [24]. Among all formulations, the M2NACa hydrogel exhibited the largest increase in contact angle upon hydration at 25°C. In contrast, the 2MNACa exhibited a lack of significant hydrophobicity enhancement. This indicated that M2NACa has a superior ability to reorganize its interfacial structure and exclude water at the adhesive surface before the temperature‐responsive phase transitions. Rheological analyses were subsequently conducted to examine the viscoelastic behavior of the three adhesive hydrogels (Figure 2C,D) at 25°C and 36°C [31, 32]. The results showed that the storage modulus (G') was found to be higher than the loss modulus (G“), confirming successful gelation of M2NACa. These findings reflected the solid‐like mechanical behavior of M2NACa and the effective network formation within this hydrogel. In contrast, both 2MNACa and MNACa exhibited semi‐liquid characteristics with comparable G' and G” values. These observations indicated the presence of insufficient crosslinking and a weaker internal network. Thus, an M:N ratio of 1:2 was identified as the optimal ratio for water‐triggered gelation.

Thermosensitive phase transitions also contribute to gelation behavior in wet environments [33]. In this study, thermochromic characterization revealed that all specimens exhibited temperature‐responsive phase transitions (Figure 2E). Notably, an increase in the NIP content promoted water expulsion and network compaction at body temperature (Figure 2F). Moreover, the reversible phase transition enabled controllable gelation kinetics (Video S2) and potentially allowed for repositioning during surgical procedures. Temperature‐dependent rheological analysis further demonstrated that the adhesive hydrogels exhibited a lower critical solution temperature between 25°C and 36°C (Figure 2G). Moreover, a marked decrease in tanδ values was observed in all hydrogels above 36°C (Figure 2H). M2NACa hydrogel exhibited the lowest tanδ value, consistent with its superior solid‐like elastic properties at physiological temperature.

Oscillatory shear scanning showed that all groups exhibited G' values characteristic of a soft gel state at 25°C. Upon heating to 36°C, the G' values increased significantly (3–50‐fold), indicating the formation of a rigid polymer network (Figure 2I). The increase in mechanical strength was inversely correlated with the decrease in tanδ values (Figure S5). Among the different formulations tested, M2NACa (M:N = 1:2) exhibited the most efficient internal network reconfiguration during thermal transition. Moreover, M2NACa also demonstrated minimal swelling behavior (Figure 2J) and achieved the highest adhesive strength (Figure 2K). Therefore, the M:N ratio of 1:2 was considered optimal and selected for subsequent experiments (Figure 2L).

### 2.3 Influence of Carboxyl‐Containing Monomers on Underwater Adhesion Performance

**FIGURE 3:**
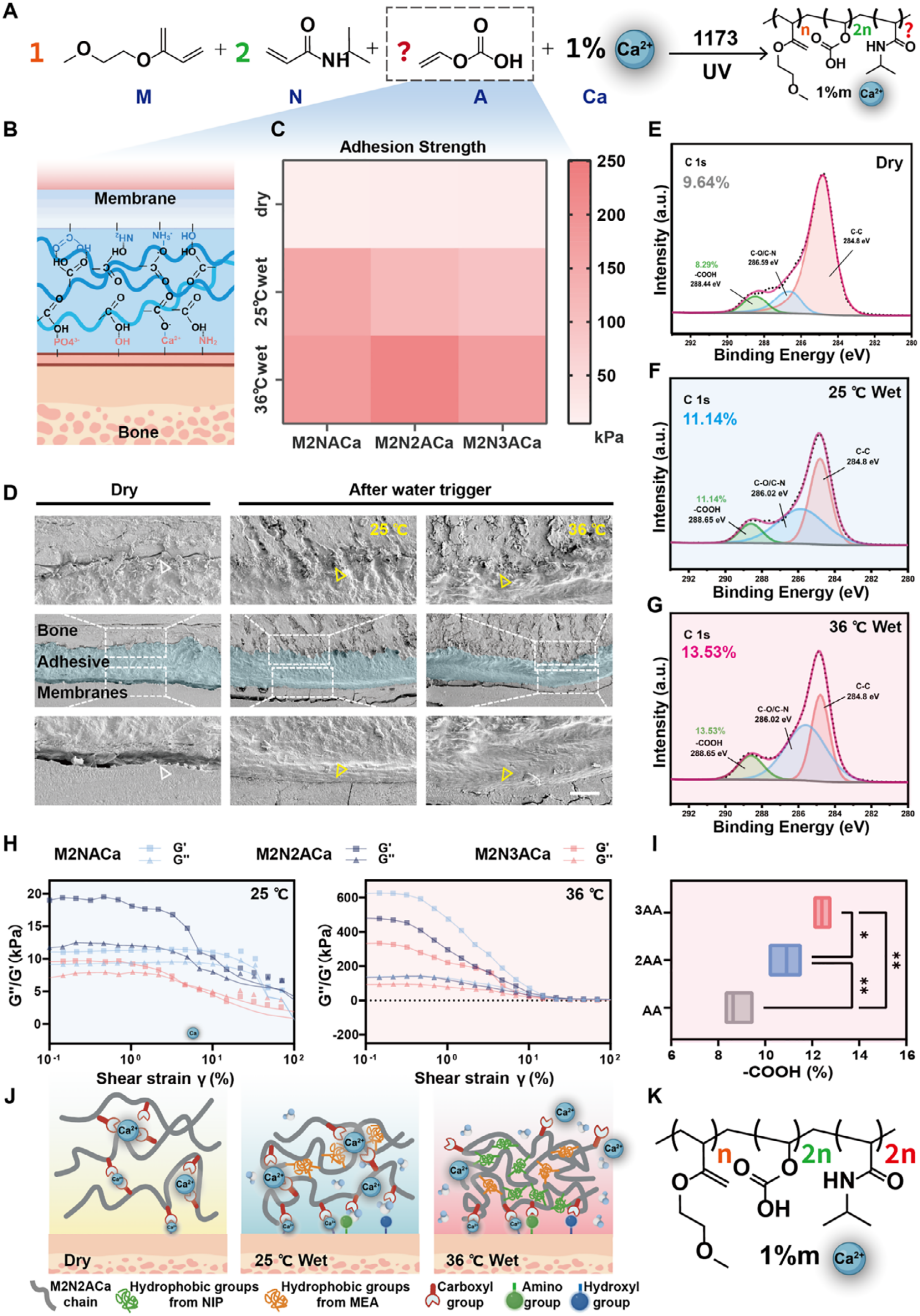
Comparison and analysis of adhesive behavior and mechanism of M2NACa, M2N2ACa, and M2N3ACa with different AA synthesis ratios. (A) Synthetic route of M2NACa, M2N2ACa, and M2N3ACa. (B) Diagrams illustrating the adhesive chemical bonds. (C) Shear strength of three adhesive hydrogels under dry conditions, 25°C wet, and 36°C wet (*n* = 6). (D) Representative SEM image of the bonding interface. The yellow triangle represents the seamless interfaces, and the white triangle represents micro‐cracks. Scale bars, 200 µm. (E) XPS spectra peak‐fitting in the C1s region of M2N2ACa. (F, G) Peak‐fitting of XPS spectra within the C1s region for lyophilized M2N2ACa solidified using 25°C water and dried M2N2ACa solidified with 37°C water. (H) The viscoelastic behavior of M2NACa, M2N2ACa, and M2N3ACa at 25°C and 36°C. (I) Comparison of ─COOH content in the surfaces of M2NACa (AA), M2N2ACa (2AA), and M2N3ACa (3AA) solidified with 37°C water, from XPS C 1s peak fitting (*n* = 3). (J) Screening results of the AA Synthesis Ratio. (K) Illustration depicting the cohesion of inner structure and adhesive interacting with bone tissue under different conditions. Data shown as mean ± standard deviation (*n* ≥ 3; ^*^ *p* < 0.05, ^**^ *p* < 0.005). The different background colors in the figure represent different experimental temperatures (Light blue represents 25°C, light red represents 36°C). Schematic generated with BioRender.

The adhesive capacity of hydrogels depends on the balanced interplay between internal cohesion and interfacial adhesion. Cohesion arises from the gelation behavior of a material, but interfacial bonding is equally essential for achieving durable adhesion under wet conditions [34, 35]. Hence, the AA ratio was systematically varied to investigate the contribution of adhesive functional groups on underwater adhesion performance (Figure 3A).

Adhesion to bone and membrane surfaces was mediated through hydrogen bonding and ionic interactions involving the carboxyl (─COOH) groups of AA (Figure 3B). Adhesion strength tests conducted under dry, 25°C wet, and 36°C wet conditions (Figure 3C) revealed a consistent trend among all AA‐containing formulations. Specifically, the adhesion strength was the highest under wet conditions at 36°C, followed by wet conditions at 25°C, with the weakest adhesion occurring under dry conditions. This confirmed that wet physiological environments can optimize the adhesive interactions of the hydrogel. However, the increase in AA content did not enhance adhesion performance linearly. The M2N2ACa hydrogel demonstrated the highest adhesive strength under wet conditions at 36°C, demonstrating that it possesses an optimal balance of functional group exposure and network stability.

Therefore, the wet adhesion mechanisms of the M2N2ACa hydrogel were analyzed in detail. Scanning electron microscopy (SEM) images (Figure 3D) revealed microcracks at the adhesive interface of M2N2ACa under dry conditions, disrupting substrate contact. In contrast, specimens cured at 36°C in wet environments formed compact, seamless interfaces. This finding was consistent with the observed modulus enhancement of the hydrogel (Figure 2I,J) and indicated the potential of tight bonding with both membrane and bone surfaces.

Surface compositional analysis using x‐ray photoelectron spectroscopy (XPS) revealed the increased surface exposure of carboxyl groups (288.8 eV) at higher temperatures in wet conditions (dry: 9.64%, 25°C wet: 11.14%, 36°C wet: 13.53%) (Figure 3E–G). These results suggested that the water‐triggered and thermally induced phase transition facilitates the removal of the interfacial hydration layer to increase surface ─COOH exposure.

Although carboxyl group exposure increased with AA concentration under wet conditions at 36°C, as detected by XPS (Figure 3I), adhesion strength followed the opposite trend (Figure 3C). This indicated that excessive AA compromises the cohesive integrity of the hydrogel. Rheological characterization supported this observation (Figure 3H). As the AA ratio increased from M2N2ACa to M2N3ACa, G' decreased and tanδ increased, reflecting greater energy dissipation and reduced network elasticity [36]. This weakening could be attributed to the hydrophilic nature of the excess ─COOH groups derived from the AA monomer, which could disrupt the hydrophobic interactions critical for the formation of a thermosensitive network [37]. Ultimately, adhesion strength is governed by the equilibrium between internal cohesive strength and interfacial bonding, as illustrated in Figure 3J. Hence, M2N2ACa (M:N:A = 1:2:2) was selected as the optimized formulation for subsequent experiments (Figure 3K).

### 2.4 In Vitro and In Vivo Biocompatibility

**FIGURE 4:**
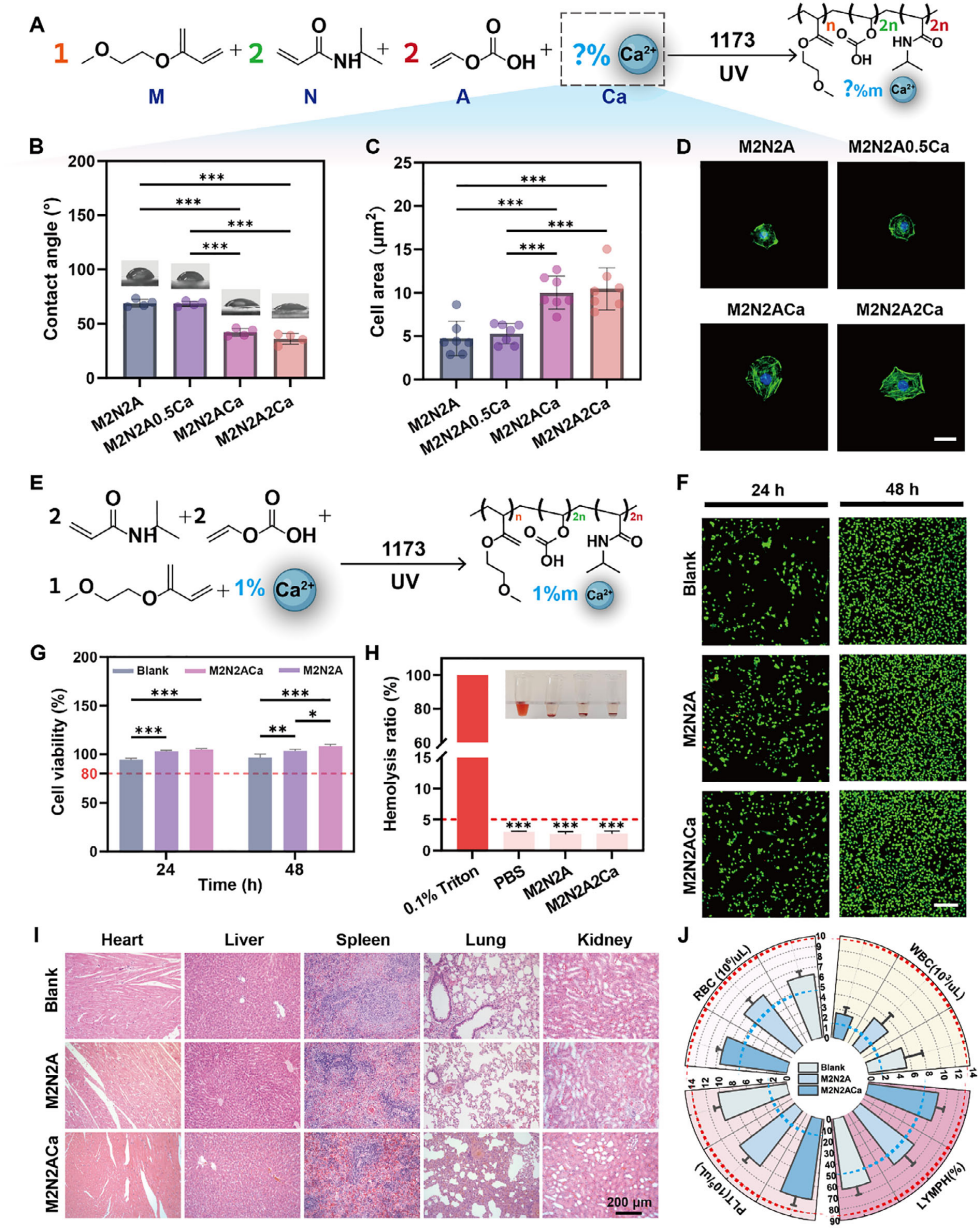
Biocompatibility of M2N2A, M2N2A0.5Ca, M2N2ACa, and M2N2A2Ca. (A) Synthetic route of M2N2A, M2N2A0.5Ca, M2N2ACa, and M2N2A2Ca. (B) The representative images and water contact angles of M2N2A, M2N2A0.5Ca, M2N2ACa, and M2N2A2Ca at 36°C (*n* = 4). (C) Statistics of the cells' spreading area (*n* = 7). (D) Representative fluorescence images of MC‐3T3 cell adhesion on the M2N2A, M2N2A0.5Ca, M2N2ACa, and M2N2A2Ca. Scale bar: 50 µm. (E) Screening results of the Ca Synthesis Ratio. (F) Live/Dead staining of cells treated with extracts of the bulk M2N2ACa (10 mg mL^−1^) for 24 and 48 h (*n* = 3). Scale bar: 100 µm. (G) Cell viability determined by co‐cultivating with the M2N2ACa extracts for 24 and 48 h (*n* = 6). M2N2A extract was designated as the control, while the tissue culture plate served as the blank control. (H) Hemolysis ratio of M2N2ACa (*n* = 6). Triton X‐100 functioned as the positive control, whereas PBS was utilized as the negative control. The red dotted line represents 5% hemolysis ratio. (I) The representative H&E staining of the main organs after the M2N2ACa subcutaneous implantation for 14 days. Scale bar: 200 µm. (J) Blood routine of rats after M2N2ACa subcutaneous implantation for 24 h (*n* = 3). Data presented as mean ± standard deviation (*n* ≥ 3; ^ns^ *p* > 0.05, ^*^ *p* < 0.05, ^**^ *p* < 0.005, ^***^ *p* < 0.001). Schematic generated with BioRender.

The hydrophilicity of materials considerably affects their impact on cellular behavior [38]. Because Ca^2+^ significantly influences the hydrophilicity of adhesive hydrogels [39], hydrogels with varying Ca^2+^ concentrations were prepared: M2N2A0.5Ca, M2N2ACa, and M2N2A2Ca (0.5%, 1%, and 2% Ca, respectively) (Figure 4A). The effects of Ca^2+^ concentration on surface wettability and cell adhesion were systematically evaluated.

As shown in Figure 4B–D, higher Ca^2+^ concentrations increased the hydrophilicity of the hydrogel and improved cell adhesion. Although adhesion properties were similar among M2N2A, M2N2A0.5Ca, and M2N2ACa, they declined significantly at the highest Ca^2+^ concentration tested in M2N2A2Ca (Figure S6). This could be because excessive hydrophilicity weakened interfacial interactions in this hydrogel. Based on these findings, 1% Ca^2+^ was selected for subsequent experiments (Figure 4E).

Good biocompatibility is a prerequisite for the in vivo application of barrier membrane adhesives [5]. Hence, CCK‐8 assays and live/dead staining were performed to assess hydrogel cytotoxicity. As shown in Figure 4F,G, Figures S7 and S8A, cell viability exceeded 80% across all groups, and almost no dead cells were detected after hydrogel treatment. These results confirmed the cytocompatibility of the hydrogels. Additionally, the hydrogels did not induce macrophage polarization and maintained a neutral immunomodulatory profile. These findings indicated that the hydrogels have negligible negative effects on the local immune microenvironment (Figure S8B–E).

In addition, because the adhesive inevitably comes into contact with blood during surgery, hemolysis tests were performed to evaluate the hemocompatibility of the hydrogels. Both M2N2ACa and M2N2A exhibited hemolysis rates below 5%, meeting in vivo safety requirements (Figure 4H). Furthermore, to evaluate in vivo biosafety, M2N2ACa was implanted subcutaneously in Sprague–Dawley rats. Subcutaneous tissues showed well‐organized local subcutaneous architecture and decreased inflammatory response 14 days after implantation (Figure S9). Major organs (liver, kidneys, spleen, lungs, and heart) revealed no signs of inflammation or histopathological abnormalities (Figure 4I). Moreover, complete blood count and routine blood chemistry parameters remained within normal physiological ranges, further demonstrating the biological safety of the material (Figure 4J). Finally, the degradation time of the adhesive was found to exceed 4 weeks, and sufficient bond strength was retained despite temporary loss (Figures S10 and S11), indicating the potential for longer‐term application.

Overall, M2N2ACa demonstrated excellent biocompatibility both in vitro and in vivo. Owing to its strong adhesive strength and favorable safety profile, the M2N2ACa hydrogel was selected for in vivo performance testing to evaluate its ability to stabilize graft materials.

### 2.5 Performance of M2N2ACa in Guided Bone Regeneration

**FIGURE 5:**
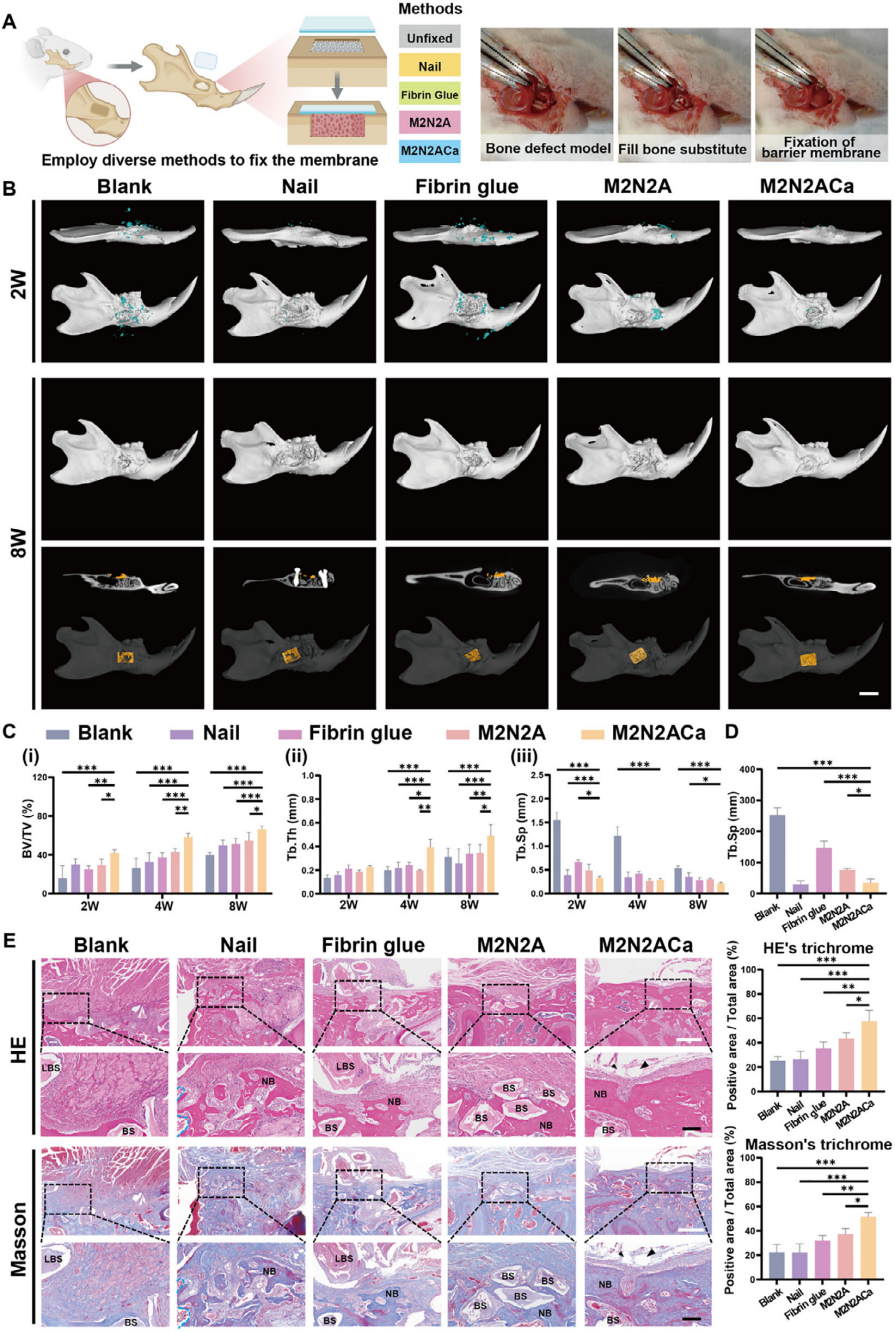
The application of M2N2ACa in vivo GBR. (A) Schematic of rat GBR with diverse methods to fix the membrane. (B) Representative micro‐computed tomography image and grey value in the defect area on the second, fourth, and eighth week after surgery. Scale bar: 4 mm. (C) Quantitative evaluation using micro‐CT of (i) the ratio of bone volume to total volume (BV/TV), (ii) trabecular bone thickness (Tb. Th), and trabecular separation (Tb. Sp) (*n* = 4). (D) Quantitative measurement of Tb. Sp in implanted bone powders within the defect area via micro‐CT (*n* = 3). (E) Histological examination and quantitative analysis of the bone defect, employing hematoxylin‐eosin and Masson trichrome staining methods, was conducted at the 8‐week time point at different magnification (BS: bone substitute; LBS: Leaked bone substitute; NB: new bone; Blue dashed line: titanium nail; Black arrow: M2N2ACa) (White scale bars = 1 mm; Black scale bar: 200 µm) (*n* = 3). Data presented as mean ± standard deviation (*n* ≥ 3; ^ns^ *p* > 0.05, ^*^ *p* < 0.05, ^**^ *p* < 0.005, ^***^ *p* < 0.001). Schematic generated with BioRender.

A dynamic periodontal bone defect model was established to replicate periodontal bone defects and the displacement of barrier membranes and bone substitutes during healing [40, 41]. Examination of the rat mandibular musculature revealed that severing the muscles attached to the masseteric ridge would cause direct friction between the masseter muscle bundles and the fossa masseterica (Figure S12), creating an ideal defect for evaluating bone substitute immobilization. After preparing the defect, four membrane fixation methods were employed (Figure 5A and Figure S13). In the negative control group, the membrane was placed over the bone substitute without fixation. Meanwhile, in the positive control groups, membrane fixation was performed using two titanium nails or using fibrin glue [42]. In the experimental groups, membrane fixation was completed using the M2N2A and M2N2ACa hydrogels (Video S3).

Three‐dimensional micro‐computed tomography (micro‐CT) reconstructions showed that the fibrin glue group and the blank group exhibited substantial leakage of bone substitutes from the bone defect, which dispersed into the surrounding soft tissues (Figure 5B). In contrast, the M2N2ACa group displayed minimal dispersion at the periphery of the defect, with retention comparable to that in the titanium nail group. Trabecular separation (Tb. Sp) analysis confirmed that M2N2ACa provided a fixation efficacy that was similar to that of titanium nails and superior to that of other fixation strategies (Figure 5D). These findings demonstrated the effective inhibition of bone substitute displacement and leakage during the early phase of osteogenesis following M2N2ACa‐mediated fixation. Interestingly, unlike the in vitro adhesion strength tests (Figure S6), in vivo assessments revealed that M2N2A was less effective than M2N2ACa at retaining bone substitute materials.

Bone regeneration outcomes were also examined using micro‐CT after 8 weeks of fixation. The M2N2ACa group achieved the highest BV/TV value (68.96%) among all groups. Notably, the BV/TV value was 39.27% in the blank group, 50.51% in the fibrin glue group, 54.00% in the M2N2A group, and 48.91% in the nail group (Figure 5C i). Trabecular thickness (Tb. Th) was also the highest in the M2N2ACa group (Figure 5C ii), while trabecular separation decreased over time when compared with the blank and nail groups (Figure 5C iii). These findings suggested that bone substitute leakage compromises bone augmentation and that M2N2ACa effectively improves outcomes.

Histological examination supported the results of the imaging studies. Hematoxylin and eosin (H&E) staining revealed more extensive new bone formation in the M2N2ACa group, while the other groups showed limited bone regeneration at 8 weeks. In the fibrin glue group, bone substitutes leaked from the defect site, accompanied by poor new bone formation. This may be attributed to the weak adhesive strength of fibrin glue, which failed to stabilize the bone substitute (Figure 5E). Quantitative analysis further confirmed that the M2N2ACa group exhibited a significantly larger area of newly formed bone within the defect region than the other groups. Masson's trichrome staining subsequently confirmed that M2N2ACa promoted a more continuous trabecular architecture and higher bone density.

Although our previous experiments indicated that M2N2ACa and calcium‐free M2N2A have comparable membrane adhesive strength, their in vivo performance for bone substitute fixation differed. This suggested that factors other than membrane adhesion were involved in in vivo bone substitute fixation. One possible explanation was that blood clots function as internal scaffolds for bone substitute fixation [7, 43, 44, 45]. The release of Ca^2+^ from M2N2ACa could influence this process by modifying blood clot properties [46].

### 2.6 Effects of M2N2ACa on the Blood Clot‐Mediated Fixation of Bone Substitutes

**FIGURE 6:**
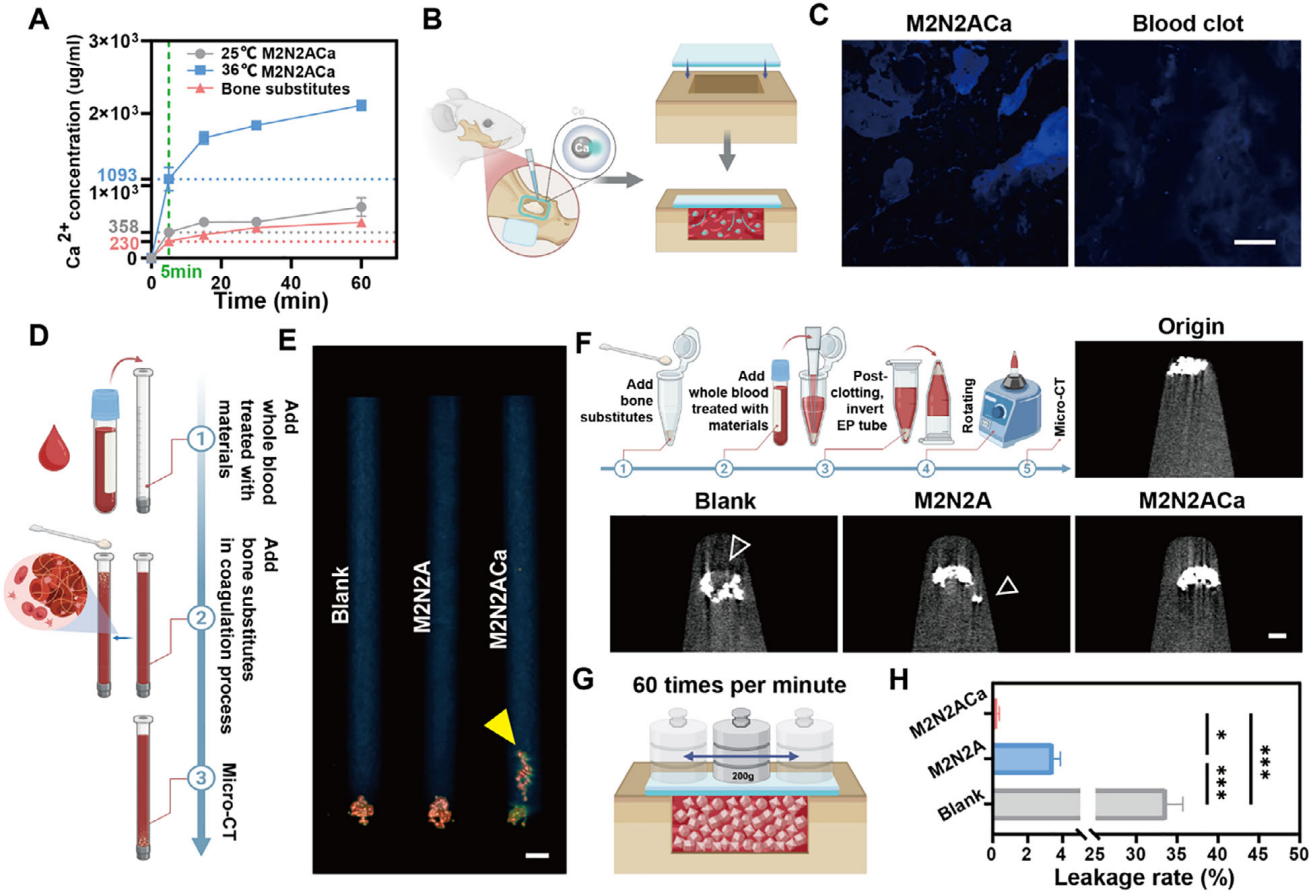
Stabilization efficacy of M2N2ACa‐modified blood clot on bone substitutes. (A) ICP‐MS analysis of Ca^2+^ release from M2N2ACa at 36°C and 25°C, compared to bone substitutes. (B) Schematic of rat GBR by using fluorophore‐conjugated Ca^2+^ M2N2ACa. (C) Representative images of fluorophore‐conjugated Ca^2+^ in M2N2ACa and blood clot. Scale bar: 100 µm. (D) Schematic of the dynamic fixation of bone substitutes with a blood clot. (E) Micro‐CT images of bone substitute location in blood clots. The yellow triangle represents the stable bone powder. Scale bar: 5 mm. (F) Illustration of the ability of blood clots to fix bone substitutes after coagulation and representative CT images of bone substitutes distribution. The white triangle represents the dispersed bone powder. Scale bar: 200 µm. (G) Schematic illustration of biomechanical loading simulation in the defect site after GBR. (H) Quantitative analysis of bone substitute leakage (*n* = 3). Data shown as mean ± standard deviation (*n* = 3; ^*^ *p* < 0.05, ^***^ *p* < 0.001). Schematic generated with BioRender.

To explore whether M2N2ACa stabilizes bone substitute materials through blood clot regulation, its ability to release Ca^2+^ was first evaluated under simulated physiological conditions using inductively coupled plasma‐mass spectrometry (ICP‐MS). As shown in Figure 6A, M2N2ACa produced burst Ca^2+^ release within 5 min at 36°C, whereas Ca^2+^ release at 25°C was minimal and gradual. This indicated that Ca^2+^ release is temperature‐dependent and that there is little concern about Ca^2+^ loss during storage. Moreover, its Ca^2+^ release capacity was greater than that of bone substitute materials. In the rat GBR model, fluorophore‐conjugated Ca^2+^ released from M2N2ACa was detected within the blood clot at the defect site, confirming that the released Ca^2+^ is incorporated into blood clots (Figure 6B,C).

The fixation of bone substitute materials during clot formation was subsequently examined. Micro‐CT revealed that M2N2ACa enhanced the stability of bone substitute materials when compared with blank and M2N2A treatment (Figure 6D,E). The influence of the clot itself on bone substitute fixation was further tested by subjecting the samples to vortex forces. Notably, the bone substitute within M2N2ACa‐treated clots remained more aggregated, whereas the M2N2A group showed a similar level of dispersion as the blank group (Figure 6F). Collectively, these findings indicated that M2N2ACa may promote the fixation effect of blood clots on bone substitutes. To simulate the mechanical forces that occur during oral function, a weighted friction test was applied to the membranes (Figure 6G,H). Bone substitute retention was found to be greater in the M2N2ACa group than in the M2N2A group. This finding confirmed that M2N2ACa is capable of stabilizing bone substitute materials by releasing Ca^2+^ to form a more stable blood clot.

Finally, the assessment of coagulation parameters indicated that M2N2ACa promoted clotting via the intrinsic pathway (Figure S14). Further analysis confirmed it also enhanced thrombin activity (Figure S15). These findings suggest its potential role in accelerating clot formation, thereby supporting the fixation of bone substitute materials. Overall, these findings suggested that M2N2ACa enhances blood clot stability around bone substitutes, although the precise mechanisms warrant further investigation.

### 2.7 Effects of M2N2ACa on Blood Clot Architecture and Mechanical Stability

Blood clots are dynamic, three‐dimensional biomechanical structures that serve as temporary scaffolds for stabilizing bone substitutes [47]. The stiffness and toughness of blood clots are critical for maintaining the initial stability of bone substitute materials.

**FIGURE 7:**
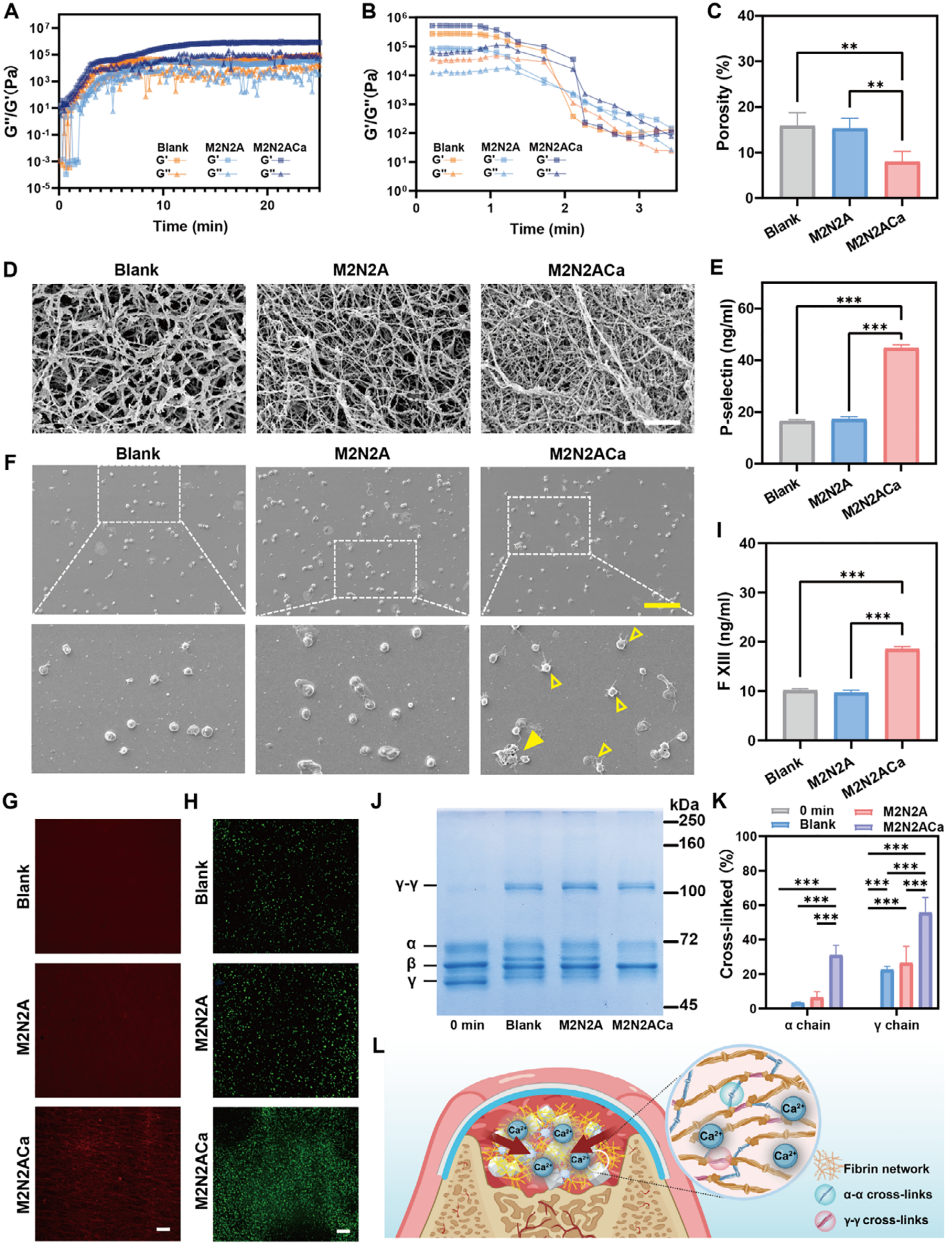
The interplay between Ca^2+^ liberated by M2N2ACa and the assembly structure of blood clots. (A) Storage and loss moduli of blood clot co‐assembling with M2N2ACa, M2N2A, and CaCl_2_. (B) The modulus of the completely gelated blood clot and the range of toughness. (C, D) SEM images show fibrin clot co‐assembling with M2N2ACa, M2N2A, and CaCl_2_, with porosity (*n* = 4) analyzed for each. Scale bar: 2 µm. (E, F) SEM shows platelet adhesion (solid yellow arrows) and activation (hollow yellow arrows) in M2N2ACa and M2N2A extracts, with ELISA statistical analysis of P‐selectin content in serum (*n* = 3). Scale bar: 20 µm. (G) Representative images of the fibrin network. Scale bar: 20 µm. (H) Representative images showing platelets in an aggregated and activated state, visualized using fluorescently labeled P‐selectin. Scale bar: 20 µm. (I) ELISA statistical analysis of FXIII content in serum treated with M2N2ACa and M2N2A extracts (*n* = 3). (J) SDS‐PAGE assessment of the extent of cross‐linking in fibrin clots treated with M2N2ACa and M2N2A extracts. (K) Densitometric analysis of the cross‐linking extent of α and γ chains, with band intensities standardized in relation to the β chain (*n* = 3). (L) Mechanistic illustration of blood clot network reinforcement in a physiological setting. Data shown as mean ± standard deviation (*n* ≥ 3; ^**^ *p* < 0.005, ^***^ *p* < 0.001). Schematic generated with BioRender.

In this study, rheological testing demonstrated that M2N2ACa accelerated clot formation and increased matrix stiffness, as indicated by higher G’ values after stabilization (Figure 7A). Oscillatory scanning further revealed that M2N2ACa can improve clot toughness, making clots more resistant to external forces (Figure 7B). These findings indicated that M2N2ACa enhances the mechanical properties of blood clots to increase the stability of bone substitutes.

The fibrin network constitutes the main mechanical framework of the blood clot. Therefore, we studied its structural organization using SEM [43, 48]. The M2N2ACa group exhibited a lower fibrin mesh porosity of 8% and a higher network density than the blank and M2N2A groups (Figure 7C,D). This densification may lead to greater clot stiffness without impairing cell migration (Figure S16). These results suggested that mechanical reinforcement does not interfere with tissue repair.

Furthermore, fibrin assembly was analyzed based on fibrinogen levels and coagulation factor XIII (FXIII) activity. The M2N2ACa adhesive increased fibrinogen concentrations (Figure S14) and promoted FXIII activation, which is a key step in forming covalent cross‐links between fibrin fibers [49, 50, 51]. Incubation experiments revealed elevated FXIII levels after M2N2ACa treatment (Figure 7I). Subsequently, SDS‐PAGE analysis demonstrated higher conversion rates for both γ‐ and α‐chains in the M2N2ACa group than in the blank and M2N2A groups (Figure 7J,K), indicating more efficient fibrin cross‐linking in the M2N2ACa group.

Given that platelet activation also contributes to fibrin network remodeling via the pseudopodia‐mediated bending and shortening of fibrin fibers, which increases clot density and stiffness [48, 52]. SEM was used to examine platelets in different groups. SEM confirmed enhanced platelet activation and aggregation in the M2N2ACa group (Figure 7F). This observation was supported by the upregulation of P‐selectin expression in this group (Figure 7E). Moreover, immunofluorescence imaging (Figure 7G,H) demonstrated accelerated fibrin assembly and greater platelet aggregation and contraction in this group vs. the control group [53]. These findings highlighted the role of Ca^2+^ in promoting fibrin formation and platelet activation.

In summary, the findings revealed that M2N2ACa modifies fibrin architecture through both direct structural effects and platelet‐mediated remodeling, promoting the formation of denser, mechanically stronger clots that are more effective at stabilizing bone substitutes (Figure 7L).

## 3 Conclusion

The M2N2ACa hydrogel developed in the present work is a promising injectable adhesive for GBR, featuring controlled thermosensitive gelation and strong wet adhesion at 36°C for securing barrier membranes. This adhesive hydrogel effectively prevents bone substitute displacement from the membrane cavity via external fixation to improve bone regeneration outcomes.

Apart from providing chemical adhesion, M2N2ACa also releases Ca^2+^, which enhances bone substitute stabilization by reinforcing blood clot stiffness via the regulation of fibrin assembly and platelet activation. This internal fixation strategy strengthens clot resistance to external stress, further reducing bone substitute displacement within the membrane cavity.

Nevertheless, research on the application of the M2N2ACa adhesive remains in its infancy, and several aspects warrant further exploration. For instance, more precisely controlled Ca^2+^ release is required to optimize clot formation, and the specific Ca^2+^ signaling pathways that govern clot remodeling need to be delineated. Moreover, the current study was conducted exclusively in rat models, and the findings must thus be validated in larger, clinically relevant animal models to better predict the translational potential of M2N2ACa. Finally, future studies should also explore the long‐term degradation profile of M2N2ACa under functional loading conditions, its performance in compromised healing environments, and its possible interactions with different graft materials and different membrane types.

Despite these limitations, this study represents a notable advancement in fixation strategies for GBR. The dual fixation concept demonstrated by M2N2ACa combines external membrane adhesion with internal clot reinforcement. This represents a novel and biologically integrated strategy for advanced next‐generation barrier membrane adhesives.

## 4 Experimental Section

### 4.1 Materials

2‐methoxyethyl acrylate (MEA; product no. 408913), N‐isopropylacrylamide (NIP; product no. 731129, ≥99% purity), acrylic acid (AA; product no. 147230, containing 200 ppm MEHQ inhibitor, 99% purity), and 2‐hydroxy‐2‐methylpropiophenone (photoinitiator 1173; product no. 4056655, 97% purity) were purchased from Millipore Sigma (Burlington, MA, USA). All other chemicals and solvents were of analytical grade and were used as supplied, without further purification.

### 4.2 Preparation and Characterization of M2N2ACa Hydrogels

Adhesive hydrogel formulations containing MEA, NIP, AA, and Ca^2+^ are collectively referred to as MNACa. The naming convention is based on the molar ratio of each monomer and the mass fraction of Ca^2+^ used for crosslinking, denoted as X_1_MX_2_NX_3_AY_4_Ca. Here, X_1_, X_2_, and X_3_ represent the molar ratios of MEA, NIP, and AA, respectively. Meanwhile, Y_4_ indicates the mass percentage of Ca^2+^ introduced as a crosslinker. For example, M2N2A0.5Ca, M2N2ACa, and M2N2A2Ca refer to hydrogels containing 0.5%, 1%, and 2% Ca^2+^, respectively.

To prepare M2N2ACa, MEA (0.019 mol), NIP (0.038 mol), and AA (0.038 mol) were dissolved in 75% ethanol and mixed with photoinitiator 1173. The solution was subjected to ultraviolet irradiation under anaerobic conditions for 60 min, resulting in the formation of the M2N2A copolymer hydrogel. After polymerization, a pre‐prepared CaCl_2_ solution in 75% ethanol was added, and the two solutions were mixed thoroughly to produce the final M2N2ACa formulation.

Different hydrogel formulations were prepared by adjusting the ratios of the monomers and Ca^2+^ according to the experimental design. Infrared spectra were recorded using attenuated total reflectance‐fourier‐transform infrared spectroscopy (ATR‐FTIR; BRUKER Vertex 70, Billerica, MA, USA). Nuclear magnetic resonance (NMR) spectra were acquired at 25°C using a 400 MHz NMR spectrometer (Bruker), with methanol as the solvent. Surface elemental composition was examined by scanning electron microscopy (SEM; Hitachi, Tokyo, Japan). The molecular weight and polydispersity index (PDI) were determined by gel permeation chromatograph (GPC) on an Agilent 1260 Infinity II system (America). Tetrahydrofuran was used as the eluent at a flow rate of 1.0 mL min^−1^, with the polystyrene standard used to generate the calibration curves.

### 4.3 Contact Angle Measurement

Adhesive hydrogel films were uniformly spread onto glass slides. For dry‐state measurements, contact angles were recorded immediately after film preparation. For hydrated‐state measurements, the films were immersed in deionized water until equilibrium swelling was achieved. The contact angles were measured using an optical contact goniometer (EasyDrop K100, Krüss GmbH, Hamburg, Germany; *n* = 4).

### 4.4 Rheological Characterization

The viscoelastic properties of the adhesive hydrogels were recorded using a rheometer (MCR 302, Anton Paar, Graz, Austria) equipped with 25‐mm parallel plates. Measurements were conducted at 25°C and 36°C. A liquid barrier was maintained around the plate's periphery to minimize water evaporation during testing.

Viscosity was evaluated across shear rates ranging from 0.1 to 100 s^−1^ at 25°C. Time sweep tests were performed at 1% strain and 1 Hz frequency for a total duration of 2 min. Oscillatory frequency sweeps were conducted under 1% strain across an angular frequency range of 0.1–100 rad s^−1^. The storage modulus (G′), loss modulus (G″), and loss tangent (tanδ) were recorded to evaluate the gelation behavior and viscoelasticity of hydrogel samples [54].

### 4.5 Adhesive Performance

The shear adhesion strength between the barrier membrane (6 × 10 mm^2^) and bovine cortical bone (3 × 6 × 20 mm^3^) was evaluated using a universal testing machine (AGS‐X, Shimadzu, Japan) at a constant shear speed of 1 mm min^−1^ at 25°C and 36°C [13]. M2N2ACa was applied to the bone surface, and this was immediately followed by the placement of the barrier membrane. Each specimen was then cured for 30 min under one of three conditions: dry, submerged in water at 25°C, or submerged in water at 36°C. Adhesion strength was calculated by dividing the maximum force by the contact area. To simulate physiological conditions, after curing in simulated body fluid (SBF) or phosphate‐buffered saline (PBS), the samples were incubated in a humidified chamber for varying durations at 25°C and 36°C.

To record the microstructure of the adhesive interface, specimens were embedded in resin, sectioned, and examined using SEM. Surface chemical composition and functional group distribution were further analyzed using x‐ray photoelectron spectroscopy (XPS; K‐Alpha, Thermo Fisher Scientific, Waltham, MA, USA), focusing on the C 1s spectral region.

### 4.6 Swelling Behavior

Hydrogel swelling behavior was examined by dispensing equal volumes of adhesive hydrogel into standardized sample containers. The initial height of the liquid was measured (*n* = 3). The specimens were then immersed in deionized water at 25°C or 36°C. Final hydrogel heights were then recorded after equilibrium swelling was achieved. The diameter of the container was used as a reference to normalize height measurements.

Image analysis for height measurements was performed using Photoshop software (Adobe Inc., San Jose, CA, USA). The swelling ratio was calculated using the following formula:

$$\begin{array}{ccc} \mathrm{Swelling}\;\left(\%\right) & = & \left[\left(\mathrm{initial}\;\mathrm{height}\;-\;\mathrm{final}\;\mathrm{height}\;\mathrm{after}\;\mathrm{swelling}\right)\right.\\ & & \left./\mathrm{initial}\;\mathrm{height}\right]\times 100\% \end{array}$$

### 4.7 Biocompatibility Assessment

To assess the cell adhesion behavior of the hydrogels, MC‐3T3 cells were seeded onto hydrogel surfaces in confocal dishes at a density of 1 × 10^4^ cells mL^−1^. After 24 h of incubation, the cells were fixed and stained with Phalloidin dye (Thermo Fisher Scientific/Invitrogen) to evaluate cytoskeletal morphology and adhesion. Leachates of M2N2ACa and M2N2A were prepared by incubating the hydrogels in complete α‐Minimum Essential Medium (α‐MEM) (Thermo Fisher Scientific/Gibco) for 24 h. Bone marrow‐derived mesenchymal stem cells and RAW 264.7 macrophages were cultured in these leachates for 24 and 48 h to evaluate cytotoxicity.

Cell viability was quantified using the Cell Counting Kit‐8 (CCK‐8, MilliporeSigma) in 96‐well plates (3 × 10^3^ cells per well). Live/dead staining was performed using a calcein/propidium iodide viability assay kit (Beyotime, Shanghai, China) in 24‐well plates (8 × 10^3^ cells per well). For hemocompatibility assessments, adhesive hydrogels were incubated with red blood cell suspensions prepared using fresh blood derived from Sprague–Dawley rats, following a previously described protocol [43]. To assess the immunomodulatory properties, RAW 264.7 macrophages were co‐cultured with hydrogel leachates of hydrogel for 2 days [55]. The polarization was further confirmed by detecting the protein expression of CD86 (M1) and CD206 (M2) using immunofluorescence staining.

In vivo biocompatibility was evaluated by injecting M2N2ACa or M2N2A subcutaneously into the backs of model rats. The hydrogels were allowed to undergo gelation in situ. A sham‐operated group served as the negative control. Blood was collected from the inner canthus using capillary tubes after 24 h of implantation. Standard hematological analysis was performed using an automated analyzer (Sysmex, Kobe, Japan). The major organs (heart, lungs, liver, spleen, and kidneys) were harvested at day 14. The material specimens with surrounding tissues were harvested at days 7 and 14. All specimens obtained were then fixed and processed for histological examination.

### 4.8 Degradation Profile

Equal volumes of adhesive hydrogels were injected into Eppendorf tubes. The initial dry weight of each specimen was recorded as W_0_. Each specimen was then incubated in simulated body fluid at 37°C for 3, 5, 7, 14, 21, and 28 days. At each time‐point, the hydrogels were removed, gently rinsed, dried, and weighed to determine W_t_. The degradation percentage was calculated using the formula: Degradation (%) = [(W_0_ − W_t_) / W_0_] × 100%.

### 4.9 In Vivo Evaluation of the M2N2ACa Hydrogel

Animal experiments were conducted under protocols approved by the Institutional Animal Care and Use Committee of the Fourth Military Medical University (IACUC‐2024‐kq‐028). Eight‐week‐old male Sprague–Dawley rats (weighing approximately 300 g) were obtained from the Laboratory Animal Research Center of the Fourth Military Medical University, Xi'an, China. The animals were randomly assigned to different treatment groups (*n* = 4 per group) and anesthetized via the intraperitoneal injection of sodium pentobarbital before surgery. All animals were healthy and showed no significant differences in body weight after the surgery.

To establish the rat periodontal defect model, a surgical incision was made along the inferior border of the mandible, followed by the careful retraction of local blood vessels and nerves. The periosteum overlying the fossa masseterica was elevated using a periosteal elevator. Subsequently, a standardized bone defect (3 × 2 × 1 mm^3^) was created using a high‐speed drill under constant irrigation with sterile saline to prevent thermal damage. The defect was completely filled with bone substitute material (Bio‐Oss (0.25–1 mm), Geistlich, Switzerland), and the site was covered with a barrier membrane (Bio‐Gide, Geistlich, Switzerland, cut to a minimum size of 5 × 4 mm^2^ to fully cover the defect) pre‐coated with either M2N2ACa or M2N2A. A final rinse with saline (40°C) was performed to complete in situ gelation.

Two positive control groups were established. In the first control group, the barrier membrane was mechanically fixed using two self‐tapping titanium bone pins (1.1 mm × 4 mm). In the second control group, the barrier membrane was fixed using a porcine fibrin sealant (Evicel, Ethicon, Raritan, NJ, USA). A blank control group, in which membrane placement was performed without fixation, was also created. Closure was achieved layer‐by‐layer using 5‐0 absorbable sutures for muscle and 4‐0 non‐absorbable sutures for skin. The rats were euthanized for evaluation at 2, 4, and 8 weeks post‐surgery.

Healing outcomes were evaluated using micro‐computed tomography (micro‐CT; Inveon; Siemens Preclinical Solutions, Knoxville, TN, USA) operated at a voltage of 80 kV and a current of 70 µA. Image reconstruction was performed using AYRecon V3.5 software. Bone regeneration was quantified using gray‐level analysis via VG Studio MAX 3.5 software to derive the bone volume fraction (BV/TV), trabecular thickness (Tb. Th), and trabecular separation (Tb. Sp). To quantitatively evaluate the leakage and dispersion of the bone substitutes, the bone substitutes displaced from the defect into the surrounding tissues were monitored. Since Tb. Sp represents the average distance between radiopaque structures, the Tb. Sp values in this region served as a quantitative indicator of bone substitute dispersion. At 8 weeks post‐surgery, the mandible samples were harvested and fixed in 4% paraformaldehyde for at least 24 h. Subsequently, the samples were immersed in the EDTA demineralizing solution for 2 months. After demineralization, the specimens were embedded in paraffin, sectioned, and stained with hematoxylin and eosin (H&E) or Masson's trichrome stain.

### 4.10 Quantification of Ca^2+^ Release

Briefly, 1 mL of the M2N2ACa hydrogel was immersed in deionized water and incubated at 37°C and 25°C to examine in vitro calcium ion release. Aliquots were collected for analysis at 5, 15, 30, and 60 min. Calcium ion concentrations were quantified using inductively coupled plasma‐mass spectrometry (ICP‐MS; NexION 2000, PerkinElmer, Waltham, MA, USA) [56].

The rat periodontal defect model was used for the in vivo analysis of local Ca^2+^ release. Calcium ions were labeled with 1‐[2‐amino‐5‐(2,7‐difluoro‐6‐hydroxy‐3‐oxo‐9‐xanthenyl)phenoxy]‐2‐(2‐amino‐5‐methylphenoxy)ethane‐N,N,N′,N′‐tetraacetic acid (Fura‐2 AM), a ratiometric fluorescent dye (Thermo Fisher Scientific). The Fura‐2–labeled calcium ions were incorporated into the M2N2A hydrogel, which was then applied to the barrier membrane and placed over the bone defect. The barrier membrane and associated blood clot were retrieved after 30 min. Fluorescence imaging was conducted using a confocal fluorescence microscope (TCS SP8, Leica Microsystems, Wetzlar, Germany) to evaluate calcium ion diffusion at the tissue interface.

### 4.11 Blood Fractioning

Blood was collected from the hearts of rats using a venous collection needle and placed in BD Vacutainer EDTA‐K_2_ vacutainer tubes (catalog 1368061, Thermo Fisher Scientific) [40]. The collection volume for each animal did not exceed 10% of the circulating blood volume. Blood samples were centrifuged at 1800 rpm for 15 min to isolate platelet‐rich plasma (PRP), and the supernatant was collected. To collect platelet‐poor plasma (PPP), the blood samples were allowed to stand for 30 min and then centrifuged at 1000 ×*g* for 10 min prior to removing the upper serum layer. All blood samples, unless specified otherwise, were re‐coagulated with 2 mm CaCl_2_ mixed with the material extract solution at a 10:1 ratio. Platelets were isolated from fresh rat blood using the Rat Peripheral Blood Platelets Isolation Kit (P8570, Solarbio, Beijing, China).

### 4.12 Evaluation of Bone Substitute Fixation by Blood Clots

Material leachates of M2N2ACa and M2N2A were prepared by incubating the respective hydrogels in saline at 36°C (10 mg mL^−1^, the concentration was based on Figure S6) for 24 h. To obtain adhesive‐treated blood samples, anticoagulated whole blood or blood fractionation samples were separately mixed with the prepared material leachates at a volume ratio of 10:1. For example, material leachates were mixed with PPP to evaluate the effect of the adhesive hydrogel on clotting parameters using an automated coagulation analyzer (Rayto RAC‐030, Shenzhen, China).

To assess the impact on the fixation of bone substitute materials during the clot formation, a 1 mL syringe was used as the buffer chamber to visually observe dynamic clotting. The adhesive‐treated blood was introduced into the chamber, with normal blood serving as a control. Bone substitutes were added at the same time point after blood injection. The position of the bone substitutes within the formed blood clot was examined via micro‐CT imaging. For the mechanical stability test, equal amounts of bone substitutes were added into 1.5 mL Eppendorf tubes, followed by the injection of equal adhesive‐treated blood samples. After the blood had coagulated, the Eppendorf tubes were vortexed. The position of bone substitutes before and after vortexing was observed by micro‐CT.

To simulate the mechanical forces exerted on bone substitutes within the oral environment, the graft leakage test was performed. The experimental procedure is illustrated in Figure S17. Standardized defects (3 mm in diameter, 2 mm in depth) were created in bovine cortical bone. To mimic the bleeding environment, the defects were first filled with anticoagulated whole blood, followed by filling with bone substitutes. The initial weight of the bone substitutes was recorded as W_1_. Subsequently, the barrier membranes were fixed with M2N2ACa or M2N2A, while an unfixed group served as the Blank control. All samples were incubated at 37°C for 15 min. These specimens were then subjected to 200 cycles of reciprocal friction under weighted conditions (200 g). After the friction test, the remaining bone substitutes within the defect were weighed as W_2_. The graft leakage rate was quantified using the following formula: Leakage (%) = (W_1_ – W_2_) / W_1_ × 100%.

### 4.13 Mechanical and Morphological Analyses of Clots

Clot rheology was examined using an MCR rheometer (Anton Paar GmbH, Graz, Austria) equipped with parallel plates (25 mm). Adhesive‐treated whole blood (500 µL) was loaded at a temperature of 36°C, with the gap set to 800 µm. Time‐dependent viscoelastic behavior was recorded at 0.01% strain and 1 Hz frequency after equilibration for 60 s. Amplitude sweep tests were conducted to monitor the G′ and G″ values across a strain range of 0.01%–100% at 36°C to determine the stiffness and toughness of the clots.

Fibrin network structure and platelet aggregation were examined using SEM following clot formation with PPP and material extracts. The mean fiber network density was determined using ImageJ software (National Institute of Health, Bethesda, MD, USA). To examine cell migration within the blood clot, the material‐incubated PRP samples were allowed to fully coagulate in confocal dishes. Subsequently, cells were seeded onto the clot surface. After 1 and 2 days, the samples were imaged with a confocal laser scanning microscope (Nikon A1R, Nikon Instruments Inc., Melville, New York, USA) to track cell infiltration. Fibrin and platelets were visualized using Alexa‐Fluor 594‐labeled fibrinogen (Solarbio) and Calcein‐AM (Beyotime) [57]. The polarization and aggregation of platelets in treated PRP samples were detected using a P‐selectin probe (Sig Biotechnology, Shanghai, China) [53].

The impact of M2N2ACa on fibrin cross‐linking was examined by quantifying Factor XIII levels in treated PPP samples using an enzyme‐linked immunosorbent assay kit (Bioswamp, China). Fibrinogen (Bergalin, China, final concentration of 0.4 mg mL^−1^) was incubated with thrombin (Solarbio, final concentration of 0.25 U mL^−1^) and CaCl_2_ (5 mmol L^−1^) at 37°C for 0 and 30 min. The mixture was analyzed using an 8% Tris‐acetate gradient gel. Coomassie blue fast staining solution (Servicebio, China) was used to visualize protein bands [51].

### 4.14 Statistical Analyses

Quantitative data were expressed as the mean ± standard deviation. The Shapiro–Wilk test was used to assess normality, and homogeneity of variance was examined with a modified Levene's test. Parametric analyses were conducted when both assumptions were satisfied. The Student's *t*‐test was used for comparisons between two groups. Meanwhile, one‐way analysis of variance (ANOVA) and Tukey's post‐hoc test were used for comparisons involving three or more groups. The non‐parametric versions of the aforementioned statistical tests were used when the normality and homoscedasticity assumptions were not fulfilled. For all tests, statistical significance was pre‐set at α = 0.05. All analyses were conducted using GraphPad Prism 5 software (GraphPad Software, La Jolla, CA, USA). Statistical significance was annotated as follows: *p* < 0.05 (^*^), *p* < 0.01 (^**^), *p* < 0.001 (^***^), and not significant (ns).

## Author Contributions

**Yu‐zhu Wang**: conceptualization, methodology, software, data curation, validation, investigation, formal analysis, visualization, writing – original draft, writing – review and editing. **Gao‐peng Dang**: Writing – review and editing, conceptualization, data curation, investigation, validation, formal analysis, methodology. **Zhi‐ting Li**: investigation, validation, methodology, formal analysis, writing – review and editing, data curation, software. **Zhi‐hong Feng**: writing – review and editing, project administration, methodology, investigation, formal analysis, supervision, data curation. **Que Bai**: methodology, investigation, validation, formal analysis, project administration. **Jia‐xin Hao**: investigation, validation, writing – review and editing, formal analysis. **Xiao‐qing Cao**: investigation, validation, data curation. **Tao Ye**: funding acquisition, visualization, formal analysis, resources, project administration. **Jing Li**: visualization, formal analysis, project administration, resources. **Franklin R. Tay**: writing – review and editing, formal analysis, supervision. **Malcolm Xing**: writing – review and editing, formal analysis, project administration, supervision. **Jun‐ting Gu**: writing – review and editing, funding acquisition, project administration, conceptualization, methodology, formal analysis, supervision, resources, visualization. **Li‐na Niu**: conceptualization, formal analysis, supervision, funding acquisition, visualization, project administration, resources, writing – review and editing.

## Conflicts of Interest

The authors declare no conflicts of interest.

## Supporting information




**Supporting File 1**: advs76877‐sup‐0001‐SuppMat.docx.

**Supporting File 2**: advs76877‐sup‐0002‐VideosS1‐S3.zip.

## Data Availability

The data that support the findings of this study are available from the corresponding author upon reasonable request.

## References

[1] D. Buser , I. Urban , A. Monje , M. Kunrath , and C. Dahlin , “Guided Bone Regeneration in Implant Dentistry: Basic Principle, Progress Over 35 Years, and Recent Research Activities,” Periodontology 2000 93, no. 1 (2023): 9–25, 10.1111/prd.12539. 38194351

[2] G. Mizraji , A. Davidzohn , M. Gursoy , U. Gursoy , L. Shapira , and A. Wilensky , “Membrane Barriers for Guided Bone Regeneration: An Overview of Available Biomaterials,” Periodontology 2000 93, no. 1 (2023): 56–76, 10.1111/prd.12502. 37855164

[3] Y. Ren , L. Fan , S. Alkildani , et al., “Barrier Membranes for Guided Bone Regeneration (GBR): A Focus on Recent Advances in Collagen Membranes,” International Journal of Molecular Sciences 23, no. 23 (2022): 14987, 10.3390/ijms232314987. 36499315 PMC9735671

[4] S. Ricard‐Blum , “The Collagen Family,” Cold Spring Harbor Perspectives in Biology 3, no. 1 (2011): a004978, 10.1101/cshperspect.a004978. 21421911 PMC3003457

[5] D. L. Aprile and T. Simon‐Yarza , “Membranes for Guided Bone Regeneration: A Road From Bench to Bedside,” Advanced Healthcare Materials 9, no. 19 (2020): 2000707, 10.1002/adhm.202000707. 32864879

[6] C. H. F. Hämmerle and R. E. Jung , “Bone Augmentation by Means of Barrier Membranes,” Periodontology 2000 33, no. 1 (2003): 36–53, 10.1046/j.0906-6713.2003.03304.x. 12950840

[7] N. Donos , A. Akcali , N. Padhye , A. Sculean , and E. Calciolari , “Bone Regeneration in Implant Dentistry: Which Are the Factors Affecting the Clinical Outcome?,” Periodontology 2000 93, no. 1 (2023): 26–55, 10.1111/prd.12518. 37615306

[8] I. A. Urban , J. L. Lozada , B. Wessing , F. Suárez‐López del Amo , and H. Wang , “Vertical Bone Grafting and Periosteal Vertical Mattress Suture for the Fixation of Resorbable Membranes and Stabilization of Particulate Grafts in Horizontal Guided Bone Regeneration to Achieve More Predictable Results: A Technical Report,” The International Journal of Periodontics & Restorative Dentistry 36, no. 2 (2016): 153–159, 10.11607/prd.2627. 26901293

[9] M. Simion , U. Misitano , L. Gionso , and A. Salvato , “Treatment of Dehiscences and Fenestrations Around Dental Implants Using Resorbable and Nonresorbable Membranes Associated With Bone Autografts: A Comparative Clinical Study,” The International Journal of Oral & Maxillofacial Implants 12, no. 2 (1997): 159–167. 9109265

[10] R. V. Yotsova , G. Y. Papanchev , M. Ali , and T. Gerova‐Vatsova , “Open Barrier Membrane Technique for the Treatment of Oroantral Communications: Two Case Reports,” Cureus (2024): 63854, 10.7759/cureus.63854. PMC11297801 39100069

[11] B. Leblebicioglu and D. N. Tatakis , “Complications Following Alveolar Ridge Augmentation Procedures,” Periodontology 2000 93, no. 1 (2023): 221–235, 10.1111/prd.12509. 37489632

[12] O. Moses , S. Pitaru , Z. Artzi , and C. E. Nemcovsky , “Healing of Dehiscence‐Type Defects in Implants Placed Together With Different Barrier Membranes: A Comparative Clinical Study,” Clinical Oral Implants Research 16, no. 2 (2005): 210–219, 10.1111/j.1600-0501.2004.01100.x. 15777331

[13] Q. Li , W. He , W. Li , et al., “Band‐Aid‐Like Self‐Fixed Barrier Membranes Enable Superior Bone Augmentation,” Advanced Science 10, no. 16 (2023): 2206981, 10.1002/advs.202206981. 37029705 PMC10238180

[14] K. Xu , X. Wu , X. Zhang , and M. Xing , “Bridging Wounds: Tissue Adhesives′ Essential Mechanisms, Synthesis and Characterization, Bioinspired Adhesives and Future Perspectives,” Burns & Trauma 10 (2022): tkac033, 10.1093/burnst/tkac033. 36225327 PMC9548443

[15] A. Bal‐Ozturk , B. Cecen , M. Avci‐Adali , et al., “Tissue Adhesives: From Research to Clinical Translation,” Nano Today 36 (2021): 101049, 10.1016/j.nantod.2020.101049. 33425002 PMC7793024

[16] S. Wei , J. Ma , L. Xu , X. Gu , and X. Ma , “Biodegradable Materials for Bone Defect Repair,” Military Medical Research 7, no. 1 (2020): 54, 10.1186/s40779-020-00280-6. 33172503 PMC7653714

[17] M. L. R. Rezende , P. O. Cunha , C. A. Damante , A. C. P. Santana , S. L. A. Greghi , and M. S. R. Zangrando , “Cyanoacrylate Adhesive as an Alternative Tool for Membrane Fixation in Guided Tissue Regeneration,” The Journal of Contemporary Dental Practice 16, no. 6 (2015): 512–518, 10.5005/jp-journals-10024-1714. 26323456

[18] H. Yuan , J. Wang , and W. Cui , “Biologically Controllable Adhesion Interfaces,” Science Bulletin 70, no. 7 (2025): 1013–1015, 10.1016/j.scib.2024.10.029. 39488452

[19] J. Yang , R. Bai , and Z. Suo , “Topological Adhesion of Wet Materials,” Advanced Materials 30, no. 25 (2018): 1800671, 10.1002/adma.201800671. 29726051

[20] Z. Ma , C. Bourquard , Q. Gao , et al., “Controlled Tough Bioadhesion Mediated by Ultrasound,” Science 377, no. 6607 (2022): 751–755, 10.1126/science.abn8699. 35951702

[21] Y. Liang , H. Xu , Z. Li , A. Zhangji , and B. Guo , “Bioinspired Injectable Self‐Healing Hydrogel Sealant With Fault‐Tolerant and Repeated Thermo‐Responsive Adhesion for Sutureless Post‐Wound‐Closure and Wound Healing,” Nano‐Micro Letters 14, no. 1 (2022): 185, 10.1007/s40820-022-00928-z. 36098823 PMC9470803

[22] P. Ducheyne , P. De Meester , and E. Aernoudt , “Influence of a Functional Dynamic Loading on Bone Ingrowth Into Surface Pores of Orthopedic Implants,” Journal of Biomedical Materials Research 11, no. 6 (1977): 811–838, 10.1002/jbm.820110603. 591524

[23] L. Milillo , F. Cinone , F. L. Presti , D. Lauritano , and M. Petruzzi , “The Role of Blood Clot in Guided Bone Regeneration: Biological Considerations and Clinical Applications With Titanium Foil,” Materials 14, no. 21 (2021): 6642, 10.3390/ma14216642. 34772167 PMC8587813

[24] Y. Zhao , Y. Wu , L. Wang , et al., “Bio‐Inspired Reversible Underwater Adhesive,” Nature Communications 8, no. 1 (2017): 2218, 10.1038/s41467-017-02387-2. PMC5738439 29263405

[25] Y. Ma , S. Ma , Y. Wu , et al., “Remote Control Over Underwater Dynamic Attachment/Detachment and Locomotion,” Advanced Materials 30, no. 30 (2018): 1801595, 10.1002/adma.201801595. 29921014

[26] K. Nagase , “Thermoresponsive Interfaces Obtained Using Poly(N‐isopropylacrylamide)‐Based Copolymer for Bioseparation and Tissue Engineering Applications,” Advances in Colloid and Interface Science 295 (2021): 102487, 10.1016/j.cis.2021.102487. 34314989

[27] H. Yuk , C. E. Varela , C. S. Nabzdyk , et al., “Dry Double‐Sided Tape for Adhesion of Wet Tissues and Devices,” Nature 575, no. 7781 (2019): 169–174, 10.1038/s41586-019-1710-5. 31666696

[28] J. Yu , R. Xie , M. Zhang , et al., “Molecular Architecture Regulation for the Design of Instant and Robust Underwater Adhesives,” Science Advances 9, no. 22 (2023): adg4031, 10.1126/sciadv.adg4031. PMC10413663 37267351

[29] K. C. Wu , B. R. Freedman , P. S. Kwon , et al., “A Tough Bioadhesive Hydrogel Supports Sutureless Sealing of the Dural Membrane in Porcine and Ex Vivo human Tissue,” Science Translational Medicine 16, no. 739 (2024): adj0616, 10.1126/scitranslmed.adj0616. PMC11145396 38507468

[30] B. Jia , B. Zhang , J. Li , et al., “Emerging Polymeric Materials for Treatment of Oral Diseases: Design Strategy towards a Unique Oral Environment,” Chemical Society Reviews 53, no. 7 (2024): 3273–3301, 10.1039/d3cs01039b. 38507263

[31] H. Ren , Z. Zhang , X. Cheng , Z. Zou , X. Chen , and C. He , “Injectable, Self‐Healing Hydrogel Adhesives With Firm Tissue Adhesion and On‐Demand Biodegradation for Sutureless Wound Closure,” Science Advances 9, no. 33 (2023): adh4327, 10.1126/sciadv.adh4327. PMC10431709 37585520

[32] G. K. Wang , Y. M. Yang , and D. Jia , “Programming Viscoelastic Properties in a Complexation Gel Composite by Utilizing Entropy‐Driven Topologically Frustrated Dynamical state,” Nature Communications 15, no. 1 (2024): 3569, 10.1038/s41467-024-47969-z. PMC11053056 38671020

[33] R. Pelton , “Poly(N‐isopropylacrylamide) (PNIPAM) Is Never Hydrophobic,” Journal of Colloid and Interface Science 348, no. 2 (2010): 673–674, 10.1016/j.jcis.2010.05.034. 20605160

[34] R. Song , X. Wang , M. Johnson , et al., “Enhanced Strength for Double Network Hydrogel Adhesive Through Cohesion‐Adhesion Balance,” Advanced Functional Materials 34, no. 23 (2024): 2313322, 10.1002/adfm.202313322.

[35] J.‐T. Gu , Z.‐T. Li , Y.‐Z. Wang , et al., “Bone Adhesive With Temporally‐Synchronized Degradation for Enhanced Osteointegration,” Bone Research 14, no. 1 (2026): 39, 10.1038/s41413-026-00522-8. 41946673 PMC13057037

[36] J. D. P. Valentin , X.‐H. Qin , C. Fessele , et al., “Substrate Viscosity Plays an Important Role in Bacterial Adhesion Under Fluid Flow,” Journal of Colloid and Interface Science 552 (2019): 247–257, 10.1016/j.jcis.2019.05.043. 31129296

[37] S. Duan , M. Hua , C. W. Zhang , et al., “Noncovalent Aggregation for Diverse Properties in Hydrogels: A Comprehensive Review,” Chemical Reviews 125, no. 16 (2025): 7918–7964, 10.1021/acs.chemrev.5c00069. 40472127

[38] N. Al‐Azzam and A. Alazzam , “Micropatterning of Cells via Adjusting Surface Wettability Using Plasma Treatment and Graphene Oxide Deposition,” PLoS ONE 17, no. 6 (2022): 0269914, 10.1371/journal.pone.0269914. PMC9202894 35709175

[39] J. G. Hardy , L. M. Römer , and T. R. Scheibel , “Polymeric Materials Based on Silk Proteins,” Polymer 49, no. 20 (2008): 4309–4327, 10.1016/j.polymer.2008.08.006.

[40] T. Ding , W. Kang , J. Li , L. Yu , and S. Ge , “An in Situ Tissue Engineering Scaffold With Growth Factors Combining Angiogenesis and Osteoimmunomodulatory Functions for Advanced Periodontal Bone Regeneration,” Journal of Nanobiotechnology 19, no. 1 (2021): 247, 10.1186/s12951-021-00992-4. 34404409 PMC8371786

[41] S. Kayumi , Y. Takayama , A. Yokoyama , and N. Ueda , “Effect of Bite Force in Occlusal Adjustment of Dental Implants on the Distribution of Occlusal Pressure: Comparison Among Three Bite Forces in Occlusal Adjustment,” International Journal of Implant Dentistry 1, no. 1 (2015): 14, 10.1186/s40729-015-0014-2. 27747636 PMC5005760

[42] C. Tu , A. Bajwa , A. Shi , G. Wu , and J. Wang , “Effect of Fibrin Glue on the Healing Efficacy of Deproteinized Bovine Bone and Autologous Bone in Critical‐Sized Calvarial Defects in Rats,” Clinical Oral Investigations 26, no. 3 (2022): 2491–2502, 10.1007/s00784-021-04217-8. 35091817

[43] J.‐T. Gu , K. Jiao , J. Li , et al., “Polyphosphate‐Crosslinked Collagen Scaffolds for Hemostasis and Alveolar Bone Regeneration After Tooth Extraction,” Bioactive Materials 15 (2021): 68–81, 10.1016/j.bioactmat.2021.12.019. 35386354 PMC8940764

[44] G.‐P. Dang , Y.‐Z. Wang , Y.‐F. Wang , et al., “Harnessing Blood Clot as a Native Scaffold for Orchestrating Tissue Repairs and Regeneration,” BMEMat (2026): e70101, 10.1002/bmm2.70101.

[45] X. Wang , T. Friis , V. Glatt , R. Crawford , and Y. Xiao , “Structural Properties of Fracture Haematoma: Current Status and Future Clinical Implications,” Journal of Tissue Engineering and Regenerative Medicine 11, no. 10 (2017): 2864–2875, 10.1002/term.2190. 27401283

[46] E. A. Ryan , L. F. Mockros , J. W. Weisel , and L. Lorand , “Structural Origins of Fibrin Clot Rheology,” Biophysical Journal 77, no. 5 (1999): 2813–2826, 10.1016/S0006-3495(99)77113-4. 10545379 PMC1300553

[47] H. T. Shiu , P. C. Leung , and C. H. Ko , “The Roles of Cellular and Molecular Components of a Hematoma at Early Stage of Bone Healing,” Journal of Tissue Engineering and Regenerative Medicine 12, no. 4 (2018): e1911–e1925, 10.1002/term.2622. 29207216

[48] M. M. Domingues , F. A. Carvalho , and N. C. Santos , “Nanomechanics of Blood Clot and Thrombus Formation,” Annual Review of Biophysics 51, no. 1 (2022): 201–221, 10.1146/annurev-biophys-111821-072110. 34990221

[49] I. K. Piechocka , N. A. Kurniawan , J. Grimbergen , J. Koopman , and G. H. Koenderink , “Recombinant Fibrinogen Reveals the Differential Roles of α‐ and γ‐Chain Cross‐Linking and Molecular Heterogeneity in Fibrin Clot Strain‐Stiffening,” Journal of Thrombosis and Haemostasis 15, no. 5 (2017): 938–949, 10.1111/jth.13650. 28166607

[50] M. M. Domingues , F. L. Macrae , C. Duval , et al., “Thrombin and Fibrinogen γ′ Impact Clot Structure by Marked Effects on Intrafibrillar Structure and Protofibril Packing,” Blood 127, no. 4 (2016): 487–495, 10.1182/blood-2015-06-652214. 26608329

[51] K. F. Standeven , A. M. Carter , P. J. Grant , et al., “Functional Analysis of Fibrin γ‐chain Cross‐Linking by Activated Factor XIII: Determination of a Cross‐Linking Pattern That Maximizes Clot Stiffness,” Blood 110, no. 3 (2007): 902–907, 10.1182/blood-2007-01-066837. 17435113

[52] S. J. Pathare , W. Eng , S. J. Lee , and A. K. Ramasubramanian , “Fibrin Prestress due to Platelet Aggregation and Contraction Increases Clot Stiffness,” Biophysical Reports 1, no. 2 (2021): 100022, 10.1016/j.bpr.2021.100022. 36425457 PMC9680775

[53] S. Padilla‐Lopategui , C. Ligorio , W. Bu , et al., “Biocooperative Regenerative Materials by Harnessing Blood‐Clotting and Peptide Self‐Assembly,” Advanced Materials 36, no. 52 (2024): 2407156, 10.1002/adma.202407156. 39543808 PMC11681309

[54] Y. Liu , G. Guan , Y. Li , et al., “Gelation of Highly Entangled Hydrophobic Macromolecular Fluid for Ultrastrong Underwater in Situ Fast Tissue Adhesion,” Science Advances 8, no. 20 (2022): abm9744, 10.1126/sciadv.abm9744. PMC9122319 35594348

[55] Q. Bai , Y. Wang , G. Dang , et al., “A Multifunctional Exudate‐Absorptive Patch Accelerates Burn Wound Healing via Antioxidant, Anti‐Inflammatory and Immunomodulatory Effects,” Nano Research 19, no. 8 (2026): 94908787, 10.26599/NR.2026.94908787.

[56] Y. Zhao , Y. Cai , W. Wang , et al., “Periosteum‐Bone Inspired Hierarchical Scaffold With Endogenous Piezoelectricity for Neuro‐Vascularized Bone Regeneration,” Bioactive Materials 44 (2024): 339–353, 10.1016/j.bioactmat.2024.10.020. 39512423 PMC11541236

[57] O. V. Kim , R. I. Litvinov , M. S. Alber , and J. W. Weisel , “Quantitative Structural Mechanobiology of Platelet‐Driven Blood Clot Contraction,” Nature Communications 8, no. 1 (2017): 1274, 10.1038/s41467-017-00885-x. PMC5668372 29097692

