# Supplementary material for: Chemical‐Biological Fixation for Enhanced Bone Augmentation in Guided Bone Regeneration
Source: Adv Sci (Weinh). 2026 Jul 30:e76877. Online ahead of print. doi: 10.1002/advs.76877 (PMC13423491; doi:10.1002/advs.76877)
Supplement: Supplementary file 1 — Supporting File 1: advs76877‐sup‐0001‐SuppMat.docx. [file ADVS-9999-e76877-s002.docx]

Supporting Information

**Chemical-Biological Fixation for Enhanced Bone Augmentation in Guided Bone Regeneration**

*Yu-zhu Wang^#^, Gao-peng Dang^#^, Zhi-ting Li^#^, Zhi-hong Feng, Que Bai, Jia-xin Hao, Xiao-qing Cao, Tao Ye, Jing Li, Franklin R. Tay, Malcolm Xing, Ming-Fang*, Jun-ting Gu*, Li-na Niu**

^#^ *These authors contributed equally to this work*

**Figure S1.** GPC profile of MNA.

**Figure S2.** Rheological and thermosensitive properties of the MNACa.

**Figure S3.** Digital photographs showing the water-triggered curing and adhesion process of MNACa.

**Figure S4.** Digital photographs showing MNACa remained steadily maintained in three simulated physiological wet environments.

**Figure S5.** Statistics of the loss factor of 2MNACa, MNACa and M2NACa at 25 and 36 ℃ under wet environments.

**Figure S6.** Adhesion performances of M2N2A, M2N2A0.5Ca, M2N2ACa, and M2N2A2Ca under wet environment at 36℃.

**Figure S7.** Cell viability of BSMCs cultured with M2N2ACa hydrogels assessed by CCK-8 assay.

**Figure S8.** Effect of M2N2ACa on macrophage biocompatibility and polarization.

**Figure S9.** Hematoxylin and eosin staining analysis of M2N2ACa and M2N2A implanted subcutaneous for 7 and 14 days.

**Figure S10.** Degradation ratios of M2N2ACa and M2N2A in SBF over 0-45 days.

**Figure S11.** Variation of underwater adhesion strength of M2N2ACa over time.

**Figure S12.** Digital image showing the masseter muscle bundles and fossa masseterica of the rat mandible.

**Figure S13.** Digital photos of different methods for fixing the membrane.

**Figure S14.** Influence of M2N2ACa and M2N2A on the prothrombin time (PT), activated partial thromboplastin time (APTT), thrombin time (TT), and fibrinogen (FIB).

**Figure S15.** Effect of material treatment on thrombin activity.

**Figure S16.** Cell migration within blood clots.

**Figure S17.** Schematic illustration of the graft leakage test.

**Video S1.** Good injectability and rapid temperature-sensitive gelation in aqueous environments.

**Video S2.** Reversible phase transition in response to body temperature.

**Video S3.** The membrane was immobilized by M2N2ACa in the defect model.

**
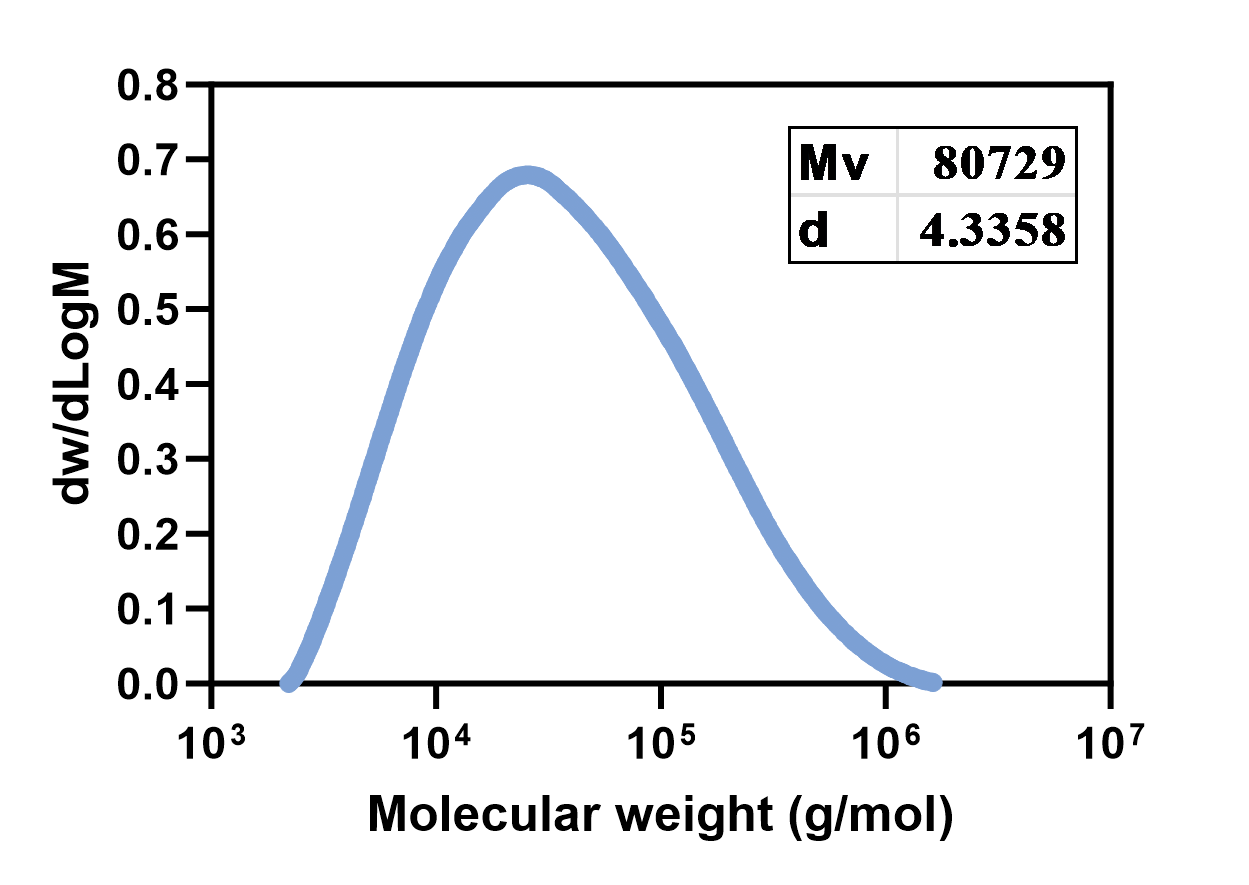
**

**Figure S1.** GPC profile of MNA.
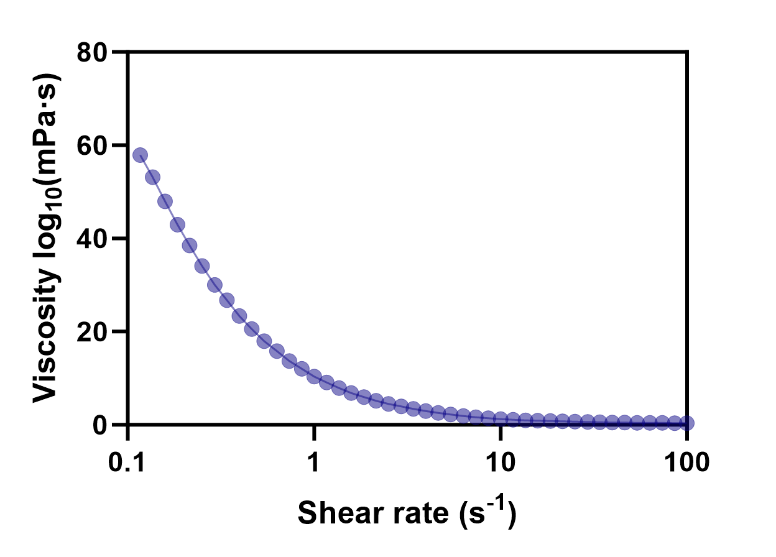

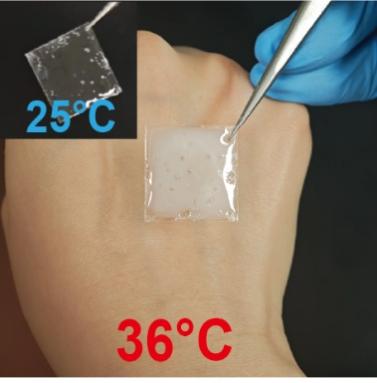

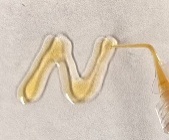


**B**

**A**

**Figure S2.** Rheological and thermosensitive properties of the MNACa. (A) Shear-thinning test. The inset showed the “A” letter formed by the injectable stained hydrogel. (B) Representative photograph of body temperature-triggered phase change.


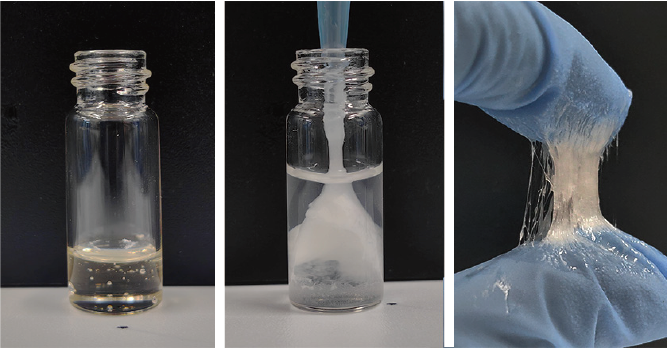


**Figure S3.** Digital photographs showing the water-triggered curing and adhesion process of MNACa**.**


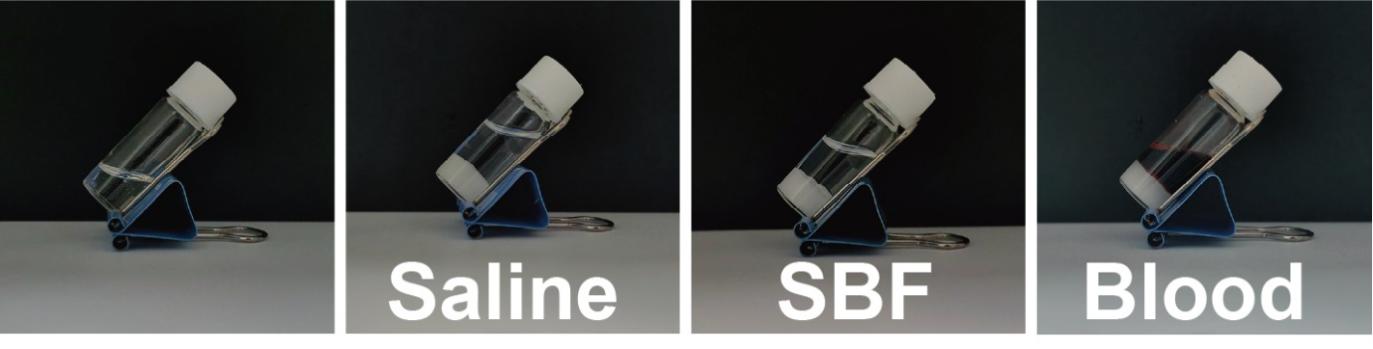


**Figure S4.** Digital photographs showing MNACa remained steadily maintained in three simulated physiological wet environments**.**


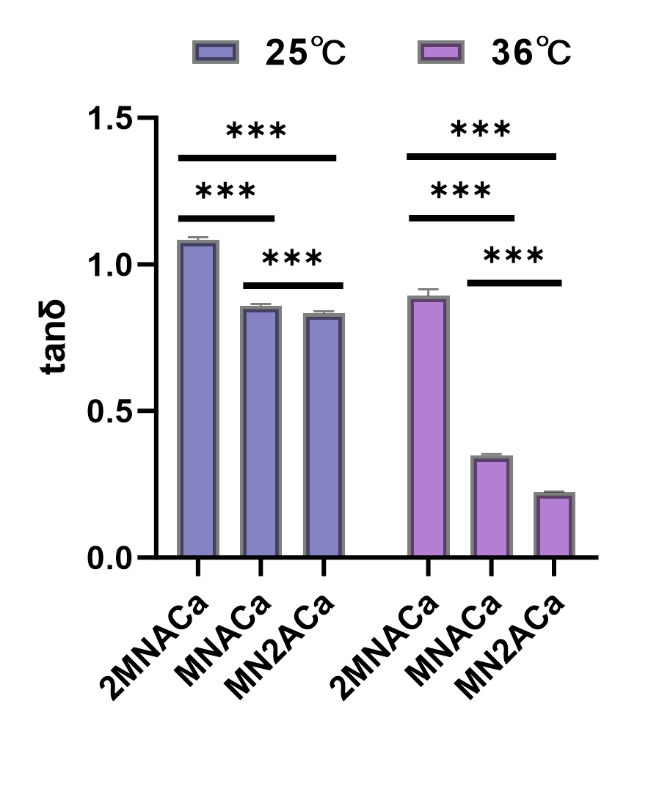


**Figure S5.** Statistics of the the loss factor of 2MNACa, MNACa, and M2NACa at 25 ℃ and 36 ℃ under wet environments. Data shown as mean ± standard deviation (n = 3; ****p* < 0.001).


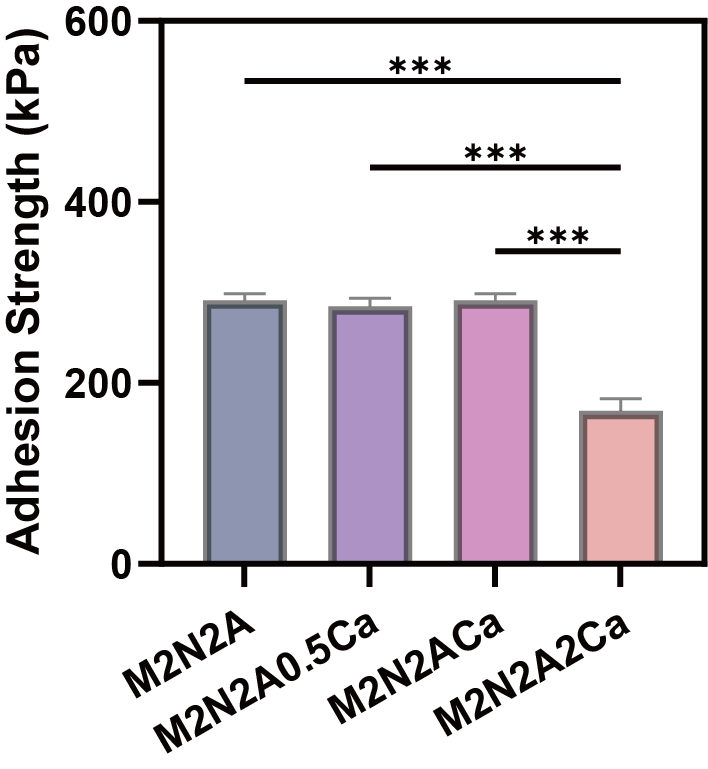


**Figure S6.** Adhesion performances of M2N2A, M2N2A0.5Ca, M2N2ACa, and M2N2A2Ca under wet conditions at 36℃ wet. Data shown as mean ± standard deviation (n = 3; ****p* < 0.001).


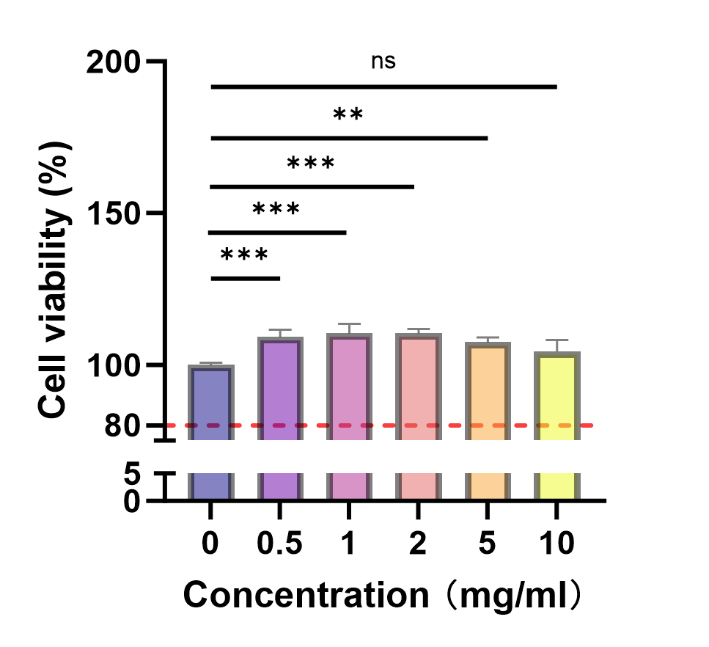


**Figure S7.** Cell viability of BSMCs cultured with M2N2ACa hydrogels assessed by CCK-8 assay. Data shown as mean ± standard deviation (n = 4; ^ns^*p* > 0.05, ***p* < 0.005, ****p* < 0.001).


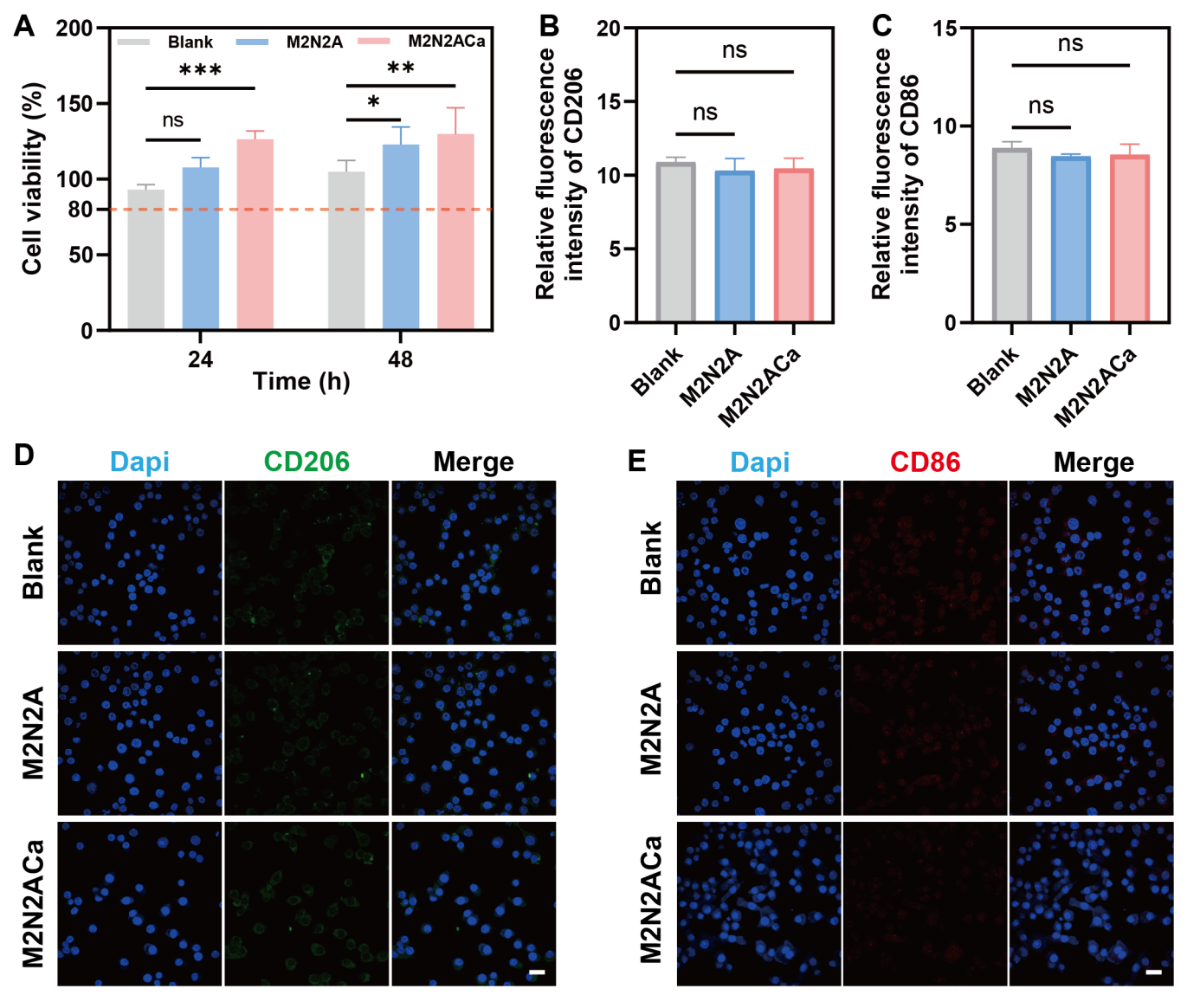


**Figure S8.** Effect of M2N2ACa on macrophage biocompatibility and polarization. (A) Cell viability of macrophages cultured with M2N2ACa hydrogels assessed by CCK-8 assay. Immunofluorescence staining and quantified fluorescence intensity of CD206 (B, D) and CD86 (C, E) in treated macrophages. Scale bar: 20um (D, E). Data shown as mean ± standard deviation (n = 5; ^ns^*p* > 0.05, **p* < 0.05, ***p* < 0.01, ****p* < 0.001).

**
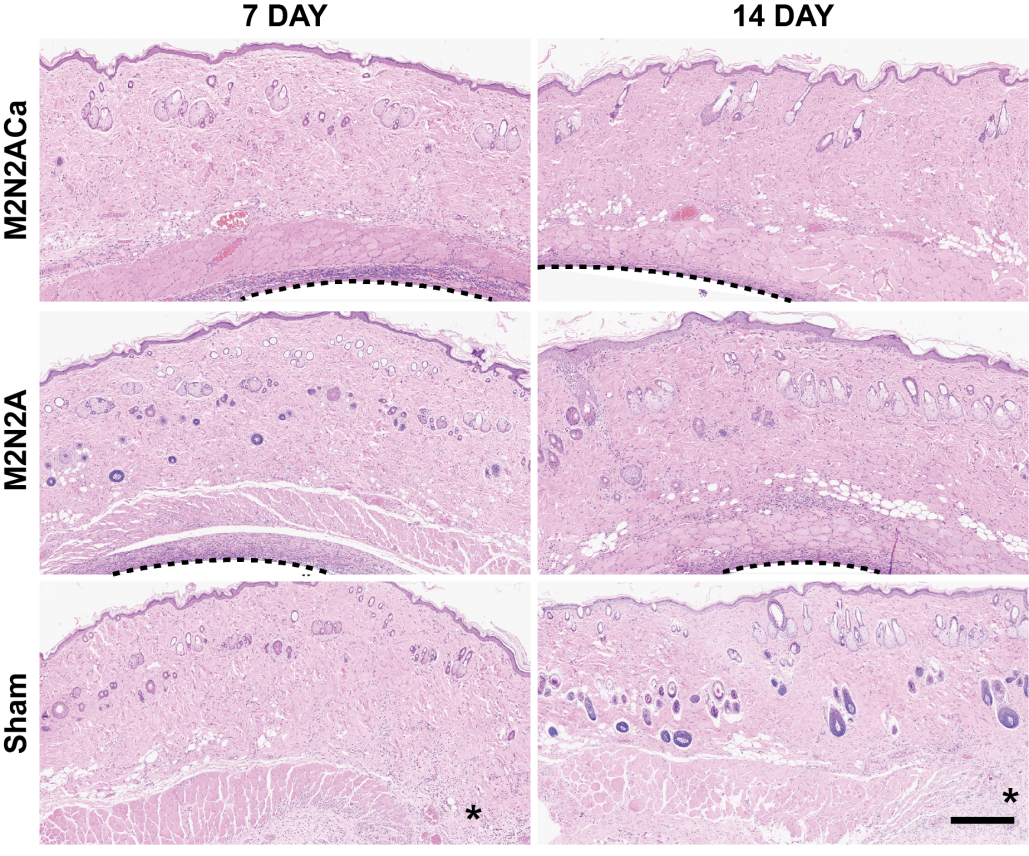
**

**Figure S9.** Hematoxylin and eosin staining analysis of M2N2ACa and M2N2A implanted subcutaneous for 7 and 14 days. Sham: sham surgery group. The dashed line represents the material infiltrated into the tissues. The black asterisk represents the trauma in the sham surgery group. Scale bar: 200 μm.


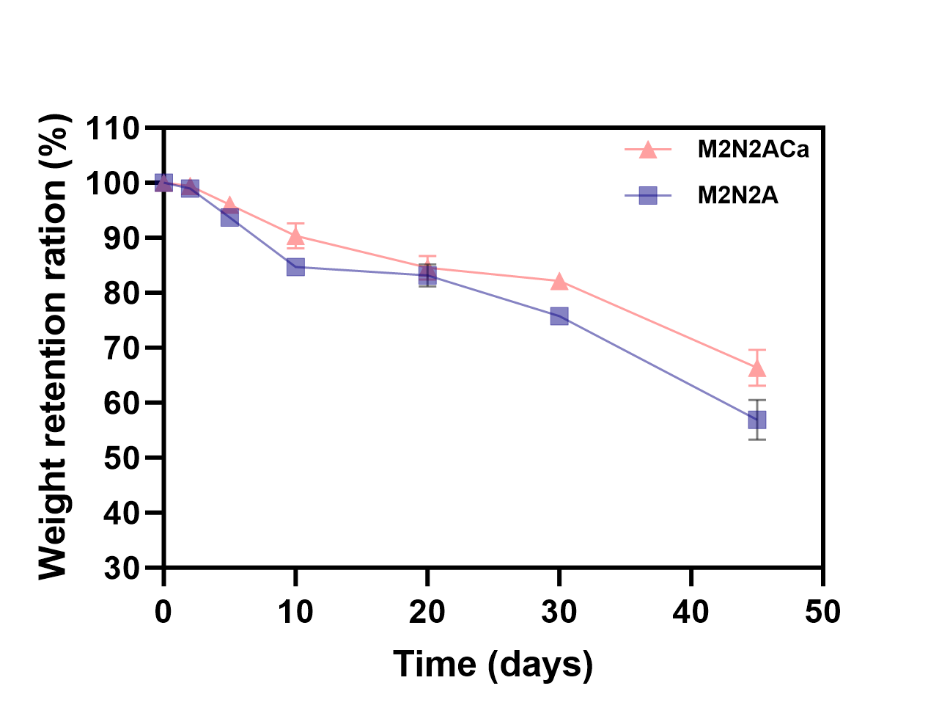


**Figure S10.** Degradation ratios of M2N2ACa and M2N2A in SBF over 0~45 days (n=5).

**
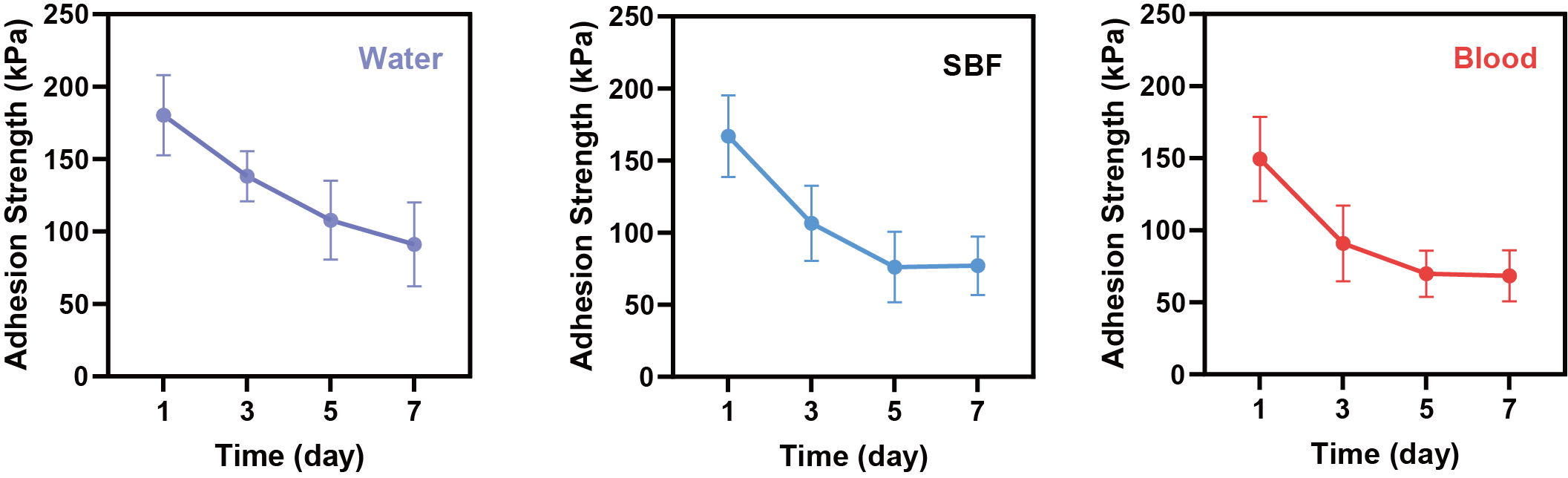
**

**Figure S11.** Variation of underwater adhesion strength of M2N2ACa over time (n = 6).


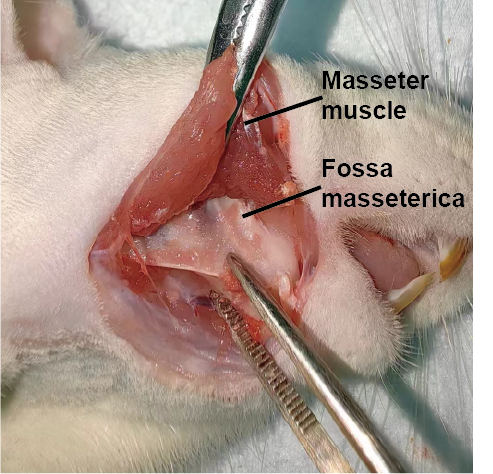


**Figure S12.** Digital image showing the masseter muscle bundles and fossa masseterica of the rat mandible.


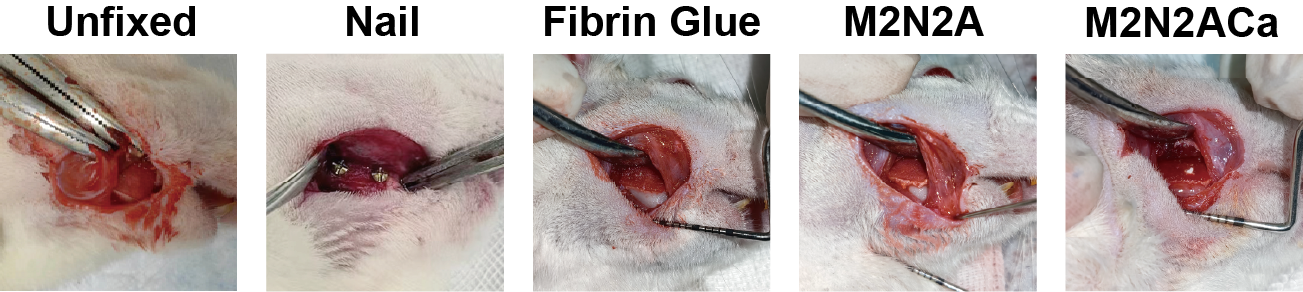


**Figure S13.** Digital photos of different methods for fixing the membrane.


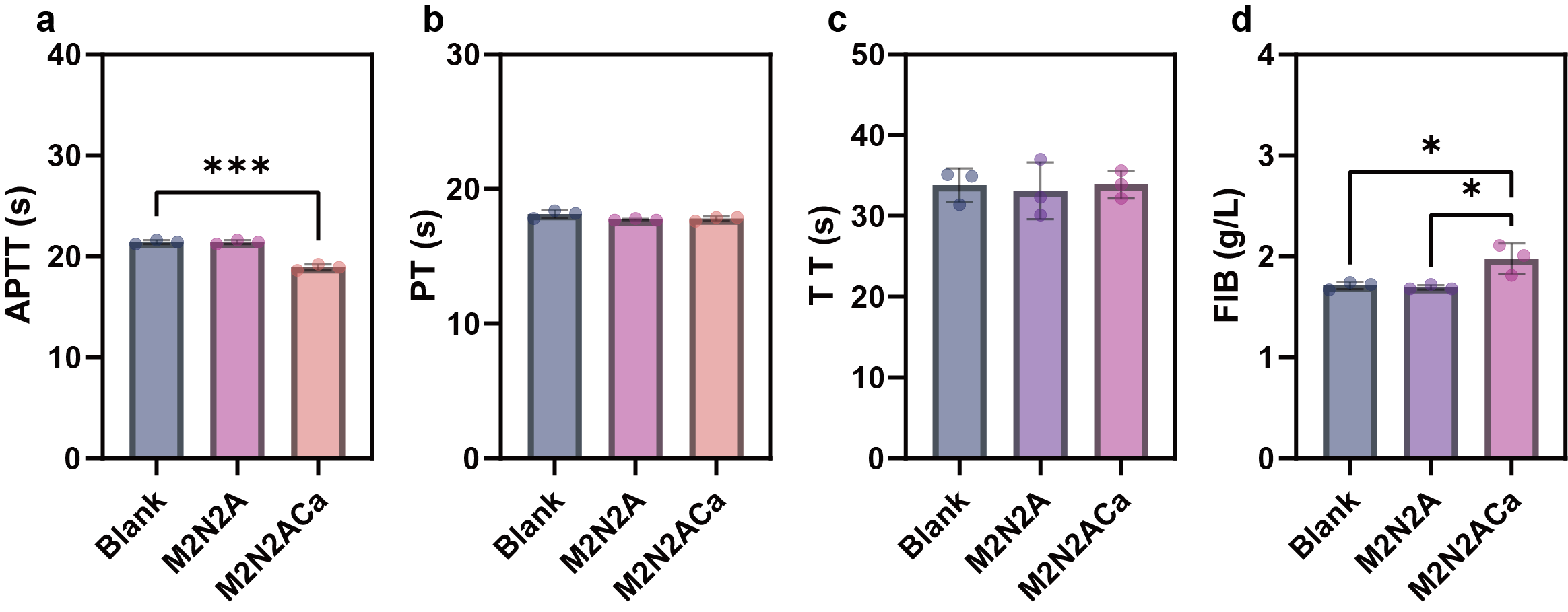


**Figure S14.** Influence of M2N2ACa and M2N2A on the prothrombin time (PT), activated partial thromboplastin time (APTT), thrombin time (TT), and fibrinogen (FIB). Data shown as mean ± standard deviation (n = 3; **p* < 0.05, ****p* < 0.001).


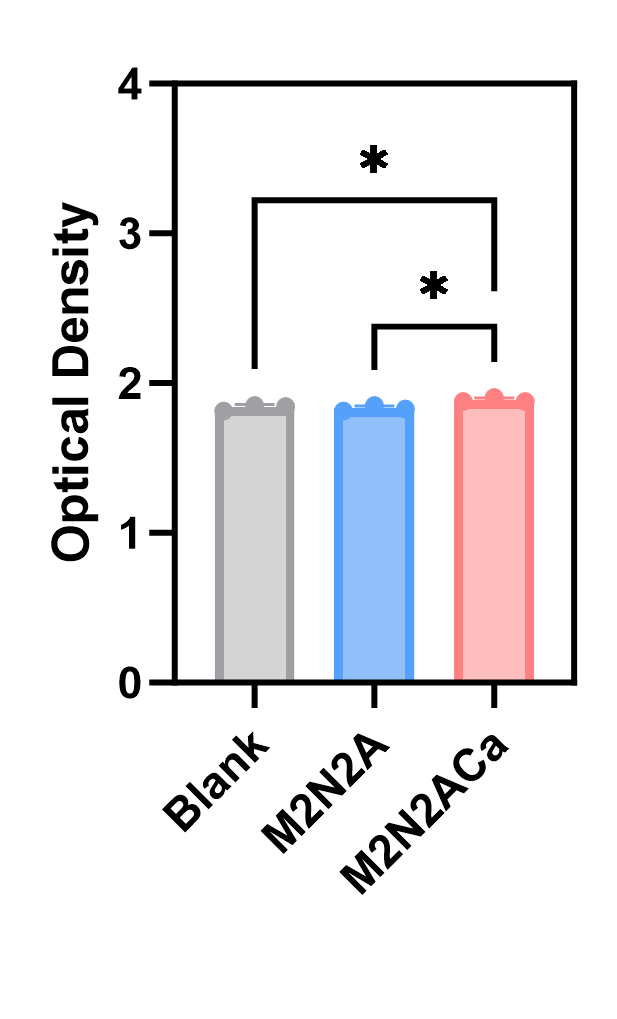


**Figure S15.** Effect of material treatment on thrombin activity (n = 3; **p* < 0.05).


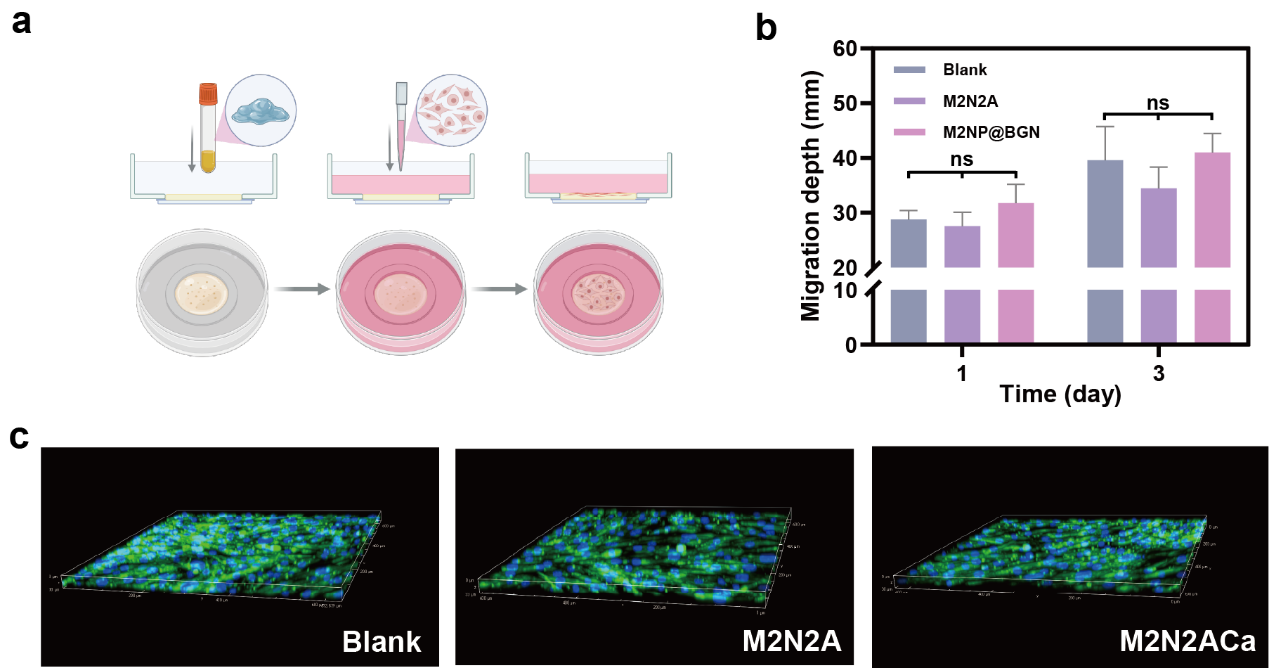


**Figure S16.** Cell migration within blood clots. (a) Schematic illustration of cell migration in a fibrin clot treated with the material for 24 and 72 h. (b-c) Statistics and analysis were performed with confocal laser scanning microscopy z-stacking. Data shown as mean ± standard deviation (n ≥ 3; ^ns^ *p* > 0.05).


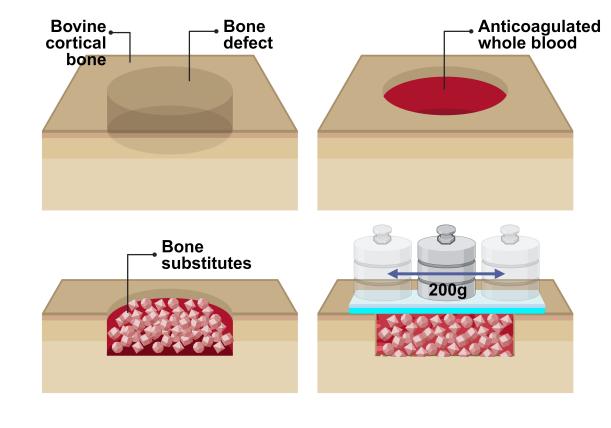


**Figure S17.** Schematic illustration of the graft leakage test.
